# Supplementary material for: Randomised controlled feasibility trial of an active communication education programme plus hearing aid provision versus hearing aid provision alone (ACE To HEAR)
Source: BMJ Open. 2021 Apr 7;11(4):e043364. doi: 10.1136/bmjopen-2020-043364 (PMC8031014; doi:10.1136/bmjopen-2020-043364)
Supplement: Supplementary data [file bmjopen-2020-043364supp002.pdf]

Supplementary material for manuscript:

A randomised controlled feasibility trial of an Active Communication Education programme plus hearing aid provision versus hearing aid provision alone (ACE To HEAR)

Figure A: Monthly recruitment to the study, by site

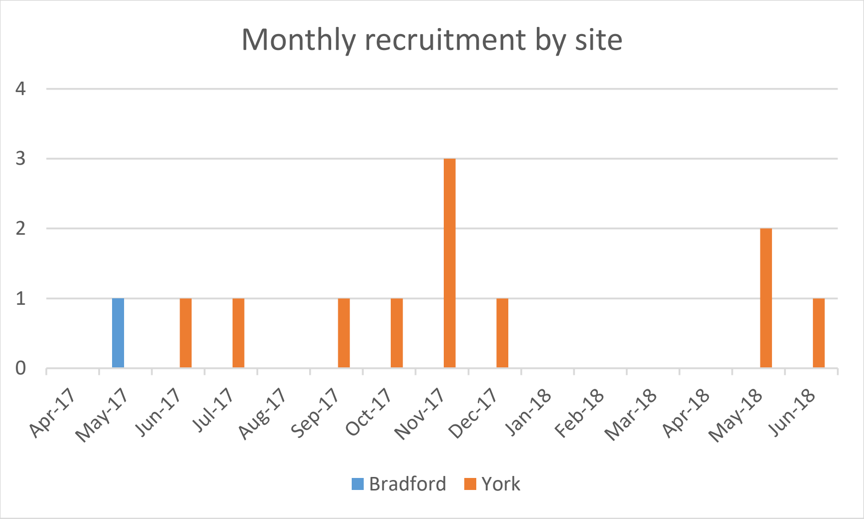


**Table A:** Hearing aid use, hearing thresholds and aetiology for the randomised participants by group and overall

| | ACE Intervention (n=4) | Control (n=4) | All (n=8) |
|---|---|---|---|
| **Average daily hearing aid use over the past two weeks:** | | | |
| None | 1 (25.0) | 0 (0.0) | 1 (12.5) |
| Less than 1 hour a day | 0 (0.0) | 0 (0.0) | 0 (0.0) |
| Between 1 and 4 hours a day | 1 (25.0) | 3 (75.0) | 4 (50.5) |
| Between 4 and 8 hours a day | 0 (0.0) | 0 (0.0) | 0 (0.0) |
| More than 8 hours a day | 2 (50.0) | 1 (25.0) | 3 (37.5) |

| | | | |
|---|---|---|---|
| **Level of help from hearing aid in the last two weeks:** | | | |
| Helped not at all | 1 (25.0) | 0 (0.0) | 1 (12.5) |
| Helped slightly | 1 (25.0) | 1 (25.0) | 2 (25.0) |
| Helped moderately | 2 (50.0) | 3 (75.0) | 5 (62.5) |
| Helped quite a lot | 0 (0.0) | 0 (0.0) | 0 (0.0) |
| Helped very much | 0 (0.0) | 0 (0.0) | 0 (0.0) |
| ***Hearing Thresholds*** | | | |
| | N=4 | N=4 | N=8 |
| ***Left Ear*** | 27.5 (9.6) | 18.8 (8.5) | 23.1 (9.6) |
| **500Hz** | 30 (15, 35) | 17.5 (10, 30) | 22.5 (10, 35) |
| Mean (SD) | | | |
| Median (min, max) | | | |
| | 40 (20.4) | 26.3 (13.8) | 33.1 (17.7) |
| **1000Hz** | 40 (15, 65) | 27.5 (10, 40) | 37.5 (10, 65) |
| Mean (SD) | | | |
| Median (min, max) | | | |
| | 61.3 (11.1) | 41.3 (8.5) | 51.3 (14.1) |
| **2000Hz** | 60 (50, 75) | 42.5 (30, 50) | 50 (30, 75) |
| Mean (SD) | | | |
| Median (min, max) | | | |
| | 75 (9.1) | 52.5 (10.4) | 63.8 (15.1) |
| **4000Hz** | 75 (65, 85) | 52.5 (40, 65) | 65 (40, 85) |
| Mean (SD) | | | |
| Median (min, max) | | | |
| | 73.8 (23.2) | 70 (9.2) | 71.9 (16.5) |
| **8000Hz** | 77.5 (45, 95) | 70 (60, 80) | 70 (45, 95) |
| Mean (SD) | | | |
| Median (min, max) | | | |
| ***Right Ear*** | | | |
| **500Hz** | N=4 | N=4 | N=8 |
| Mean (SD) | 26.3 (18.9) | 22.5 (8.7) | 24.4 (13.7) |
| Median (min, max) | 25 (10, 45) | 20 (15, 35) | 20 (10, 45) |
| **1000Hz** | | | |
| Mean (SD) | 36.3 (15.5) | 32.5 (15.5) | 34.4 (14.5) |
| Median (min, max) | 40 (15, 50) | 37.5 (10, 45) | 37.5 (10, 50) |
| **2000Hz** | | | |
| Mean (SD) | 41.3 (7.5) | 52.5 (22.2) | 46.9 (16.5) |
| Median (min, max) | 45 (30, 45) | 50 (30, 80) | 45 (30, 80) |
| **4000Hz** | | | |

| | | | |
|---|---|---|---|
| Mean (SD) | 75 (14.8) | 63.8 (19.3) | 69.4 (17.0) |
| Median (min, max) | 75 (60, 90) | 62.5 (45, 85) | 70 (45, 90) |
| **8000Hz** | | | |
| Mean (SD) | 80 (23.8) | 71.3 (25.0) | 75.6 (23.1) |
| Median (min, max) | 90 (45, 95) | 65 (50, 105) | 80 (45, 105) |
| **Aetiology, n (%)** | | | |
| Sensorineural | 3 (75.0) | 4 (100.0) | 7 (87.5) |
| Conductive | 0 (0.0) | 0 (0.0) | 0 (0.0) |
| Mixed | 1 (25.0) | 0 (0.0) | 1 (12.5) |
| Other | 0 (0.0) | 0 (0.0) | 0 (0.0) |

**Table B:** International Outcomes Inventory for Hearing Aids (IOI-HA) results for participants, by group and overall (Higher score is better, range 7-35)

| | ACE Intervention participants (n=4) | Control participants (n=4) | All participants (n=8) |
|---|---|---|---|
| **Baseline** | N=4 | N=3 | N=7 |
| Mean (SD) | 22.5 (6.6) | 25.7 (3.1) | 23.9 (5.3) |
| Median (min, max) | 23 (15, 29) | 25 (23, 29) | 25 (15, 29) |
| **Week 5** | N=3 | N=3 | N=6 |
| Mean (SD) | 23.0 (3.6) | 26.3 (4.7) | 24.7 (4.2) |
| Median (min, max) | 24 (19, 26) | 28 (21, 30) | 25 (19, 30) |
| **3 months post-randomisation** | N=3 | N=3 | N=6 |
| Mean (SD) | 20 (1.7) | 27.7 (3.5) | 23.9 (4.9) |
| Median (min, max) | 19 (19, 22) | 28 (24, 31) | 23 (19, 31) |

**Table C:** Self-Assessment of Communication (SAC) (Lower score is better, range 0 – 100)

| | ACE Intervention participants (n=4) | Control Participants (n=4) | All participants (n=8) |
|---|---|---|---|
| **Baseline** | N=4 | N=4 | N=8 |
| Mean (SD) | 65 (21.9) | 20 (7.9) | 42.5 (28.5) |
| Median (min, max) | 58.8 (47.5, 95) | 21.3 (10, 27.5) | 37.5 (10, 95) |
| **3 months post-randomisation** | N=3 | N=3 | N=6 |
| Mean (SD) | 62.5 (10.9) | 22.5 (4.3) | 42.5 (23.1) |
| Median (min, max) | 57.5 (55, 75) | 25 (17.5, 25) | 40 (17.5, 75) |

**Table D**: International Outcomes Inventory for Alternative Interventions (IOI-AI) (scored 1 – 5, higher score is better)

| | ACE intervention participants (n=4) |
|---|---|
| **Week 5** | N=3 |
| Mean (SD) | 2.6 (0.7) |
| Median (min, max) | 3 (1.9, 3.1) |
| | |
| **3 months post-randomisation** | N=2 |
| Mean (SD) | 2.6 (0.6) |
| Median (min, max) | 2.6 (2.1, 3) |

**Table E:** International Outcomes Inventory for Hearing Aids: version for Significant Others (IOI-HA-SO) results for significant others, overall (Higher score is better, range 7 - 35)

| | All SOs (n=4) |
|---|---|
| **Baseline** | N=4 |
| Mean (SD) | 23.8 (5.3) |
| Median (min, max) | 24 (17, 30) |
| | |
| **Week 5** | N=3 |
| Mean (SD) | 25.7 (3.1) |
| Median (min, max) | 25 (23, 29) |
| | |
| **3 months post-randomisation** | N=3 |
| Mean (SD) | 24 (4.0) |
| Median (min, max) | 24 (20, 28) |

**Table F:** Significant Other Scale for Hearing Disability (SOS-HEAR) (scored 0 – 4, higher scores indicate greater difficulties experienced by the SO.)

| | All SOs (n=4) |
|---|---|
| **Baseline** | N=4 |
| Mean (SD) | 1.0 (0.5) |
| Median (min, max) | 0.9 (0.5, 1.8) |
| | |
| **Week 5** | N=3 |
| Mean (SD) | 1.2 (1.0) |
| Median (min, max) | 0.8 (0.5, 2.3) |
| | |
| **3 months post-randomisation** | N=3 |
| Mean (SD) | 1.4 (1.0) |
| Median (min, max) | 1.1 (0.6, 2.4) |

**Table G:** Average health care resource use by trial arm at baseline

| | ACE | | | | TAU | | | |
|---|---|---|---|---|---|---|---|---|
| | **n** | **Mean (SD)** | **Min, Max** | **Missing** | **n** | **Mean (SD)** | **Min, Max** | **Missing** |
| **GP Visit** | 2 | 0.5 0.71 | 0 1 | 2 | 4 | 0 | 0, 0 | 0 |
| **home** | 2 | 0 (0) | 0, 0 | 2 | 4 | 0 (0) | 0, 0 | 0 |
| **Physiotherapist** | 2 | 0 (0) | 0, 0 | 2 | 4 | 0 (0) | 0, 0 | 0 |
| **Nurse** | 2 | 0 (0) | 0, 0 | 2 | 4 | 0 (0) | 0, 0 | 0 |
| **District/ community nurse** | 0 | 0 (0) | 0, 0 | | 4 | 0 (0) | 0, 0 | 0 |
| **Hearing therapist** | 2 | 1 (1.41) | 0, 2 | 2 | 3 | 0.33 (0.58) | 0, 1 | 1 |
| **Speech therapist** | 2 | 0 (0) | 0, 0 | 2 | 4 | 0 (0) | 0, 0 | 0 |
| **Counsellor** | 2 | 0 (0) | 0, 0 | 2 | 4 | 0 (0) | 0, 0 | 0 |
| **Psychologist** | 2 | 0 (0) | 0, 0 | 2 | 4 | 0 (0) | 0, 0 | 0 |
| **Psychiatrist** | 2 | 0 (0) | 0, 0 | 2 | 4 | 0 (0) | 0, 0 | 0 |
| **ENT nurse** | 2 | 0 (0) | 0, 0 | 2 | 4 | 0.75 (1.5) | 0, 3 | 0 |
| **ENT doctor** | 2 | 0 (0) | 0, 0 | 2 | 4 | 0.75 (1.5) | 0, 3 | 0 |
| **Outpatient attendance** | 2 | 1.5 (0.71) | 1 2 | 2 | 4 | 1.75 (1.71) | 0, 4 | 0 |
| **A&E attendance** | 2 | 0 (0) | 0, 0 | 2 | 4 | 0 (0) | 0, 0 | 0 |
| **Day case** | 2 | 0.5 (0.71) | 0, 1 | 2 | 4 | 0 (0) | 0, 0 | 0 |
| **In patient per night** | 2 | 0 (0) | 0, 0 | 2 | 4 | 0 (0) | 0, 0 | 0 |
| **Private physiotherapist** | 2 | 0 (0) | 0, 0 | 2 | 4 | 0 (0) | 0, 0 | 0 |
| **Private audiologist** | 2 | 0 (0) | 0, 0 | 2 | 4 | 0 (0) | 0, 0 | 0 |
| **Private osteopath** | 2 | 0 (0) | 0, 0 | 2 | 4 | 0 (0) | 0, 0 | 0 |
| **Private counsellor** | 2 | 0 (0) | 0, 0 | 2 | 4 | 0 (0) | 0, 0 | 0 |
| **Private acupuncturist** | 2 | 0 (0) | 0, 0 | 2 | 4 | 0 (0) | 0, 0 | 0 |
| **Private chiropractitioner** | 2 | 0 (0) | 0, 0 | 2 | 4 | 0 (0) | 0, 0 | 0 |
| **Private day case** | 2 | 0 (0) | 0, 0 | 2 | 4 | 0 (0) | 0, 0 | 0 |
| **Private in patient per night** | 2 | 0 (0) | 0, 0 | 2 | 4 | 0 (0) | 0, 0 | 0 |

**Table H:** Average health care resource use by trial arm at 3 months post-randomisation

| | ACE | | | | TAU | | | |
|---|---|---|---|---|---|---|---|---|
| | **n** | **Mean (SD)** | **Min, Max** | **Missing** | **n** | **Mean (SD)** | **Min, Max** | **Missing** |
| **GP Visit** | 1 | 0 (0) | 0, 0 | 3 | 3 | 0 (0) | 0, 0 | 1 |
| **home** | 1 | 0 (0) | 0, 0 | 3 | 3 | 0 (0) | 0, 0 | 1 |
| **Physiotherapist** | 1 | 0 (0) | 0, 0 | 3 | 3 | 0 (0) | 0, 0 | 1 |
| **Nurse** | 0 | - | - | 4 | 3 | 0 (0) | 0, 0 | 1 |
| **District/ community nurse** | 1 | 0 (0) | 0, 0 | 3 | 3 | 0 (0) | 0, 0 | 1 |
| **Hearing therapist** | 2 | 0.5 (0.71) | 0, 1 | 2 | 3 | 0 (0) | 0, 0 | 1 |
| **Speech therapist** | 1 | 0 (0) | 0, 0 | 3 | 3 | 0 (0) | 0, 0 | 1 |
| **Counsellor** | 1 | 0 (0) | 0, 0 | 3 | 3 | 0 (0) | 0, 0 | 1 |
| **Psychologist** | 1 | 0 (0) | 0, 0 | 3 | 3 | 0 (0) | 0, 0 | 1 |
| **Psychiatrist** | 1 | 0 (0) | 0, 0 | 3 | 3 | 0 (0) | 0, 0 | 1 |
| **ENT nurse** | 1 | 1 (-) | 1, 1 | 3 | 3 | 0 (0) | 0, 0 | 1 |
| **ENT doctor** | 1 | 0 (0) | 0, 0 | 3 | 3 | 0 (0) | 0, 0 | 1 |
| **Outpatient attendance** | 1 | 1 (-) | 1, 1 | 3 | 3 | 0 (0) | 0, 0 | 1 |
| **A&E attendance** | 1 | 0 (0) | 0, 0 | 3 | 3 | 0 (0) | 0, 0 | 1 |
| **Day case** | 1 | 0 (0) | 0, 0 | 3 | 3 | 0 (0) | 0, 0 | 1 |
| **In patient per night** | 1 | 0 (0) | 0, 0 | 3 | 3 | 0 (0) | 0, 0 | 1 |
| **Private physiotherapist** | 1 | 0 (0) | 0, 0 | 3 | 3 | 0 (0) | 0, 0 | 1 |
| **Private audiologist** | 1 | 2 (-) | 2, 2 | 3 | 3 | 0 (0) | 0, 0 | 1 |
| **Private osteopath** | 1 | 0 (0) | 0, 0 | 3 | 3 | 0 (0) | 0, 0 | 1 |
| **Private counsellor** | 1 | 0 (0) | 0, 0 | 3 | 3 | 0 (0) | 0, 0 | 1 |
| **Private acupuncturist** | 1 | 0 (0) | 0, 0 | 3 | 3 | 0 (0) | 0, 0 | 1 |
| **Private chiropractitioner** | 1 | 0 (0) | 0, 0 | 3 | 3 | 0 (0) | 0, 0 | 1 |
| **Private day case** | 1 | 0 (0) | 0, 0 | 3 | 3 | 0 (0) | 0, 0 | 1 |
| **Private in patient per night** | 1 | 0 (0) | 0, 0 | 3 | 3 | 0 (0) | 0, 0 | 1 |
