# Supplementary material for: Randomised controlled feasibility trial of an active communication education programme plus hearing aid provision versus hearing aid provision alone (ACE To HEAR)
Source: BMJ Open. 2021 Apr 7;11(4):e043364. doi: 10.1136/bmjopen-2020-043364 (PMC8031014; doi:10.1136/bmjopen-2020-043364)
Supplement: Supplementary data [file bmjopen-2020-043364supp001.pdf]

Supplementary material for manuscript:

A randomised controlled feasibility trial of an Active Communication Education programme plus hearing aid provision versus hearing aid provision alone (ACE To HEAR)

Figure A: Monthly recruitment to the study, by site

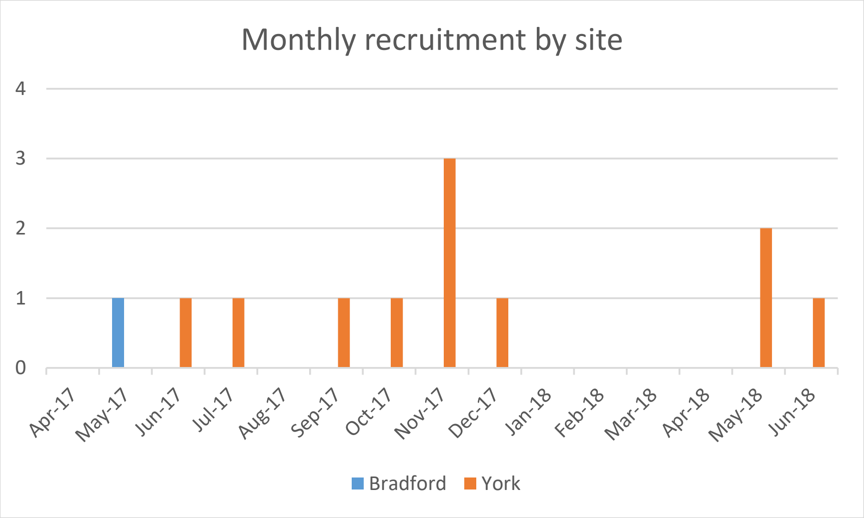

Table A: Hearing aid use, hearing thresholds and aetiology for the randomised participants by group and overall

	ACE Intervention (n=4)	Control (n=4)	All (n=8)
**Average daily hearing aid use over the past two weeks:**			
None	1 (25.0)	0 (0.0)	1 (12.5)
Less than 1 hour a day	0 (0.0)	0 (0.0)	0 (0.0)
Between 1 and 4 hours a day	1 (25.0)	3 (75.0)	4 (50.5)
Between 4 and 8 hours a day	0 (0.0)	0 (0.0)	0 (0.0)
More than 8 hours a day	2 (50.0)	1 (25.0)	3 (37.5)

**Level of help from hearing aid in the last two weeks:**			
Helped not at all	1 (25.0)	0 (0.0)	1 (12.5)
Helped slightly	1 (25.0)	1 (25.0)	2 (25.0)
Helped moderately	2 (50.0)	3 (75.0)	5 (62.5)
Helped quite a lot	0 (0.0)	0 (0.0)	0 (0.0)
Helped very much	0 (0.0)	0 (0.0)	0 (0.0)
Hearing Thresholds			
	N=4	N=4	N=8
Left Ear	27.5 (9.6)	18.8 (8.5)	23.1 (9.6)
**500Hz**	30 (15, 35)	17.5 (10, 30)	22.5 (10, 35)
Mean (SD)			
Median (min, max)			
	40 (20.4)	26.3 (13.8)	33.1 (17.7)
**1000Hz**	40 (15, 65)	27.5 (10, 40)	37.5 (10, 65)
Mean (SD)			
Median (min, max)			
	61.3 (11.1)	41.3 (8.5)	51.3 (14.1)
**2000Hz**	60 (50, 75)	42.5 (30, 50)	50 (30, 75)
Mean (SD)			
Median (min, max)			
	75 (9.1)	52.5 (10.4)	63.8 (15.1)
**4000Hz**	75 (65, 85)	52.5 (40, 65)	65 (40, 85)
Mean (SD)			
Median (min, max)			
	73.8 (23.2)	70 (9.2)	71.9 (16.5)
**8000Hz**	77.5 (45, 95)	70 (60, 80)	70 (45, 95)
Mean (SD)			
Median (min, max)			
Right Ear			
**500Hz**	N=4	N=4	N=8
Mean (SD)	26.3 (18.9)	22.5 (8.7)	24.4 (13.7)
Median (min, max)	25 (10, 45)	20 (15, 35)	20 (10, 45)
**1000Hz**			
Mean (SD)	36.3 (15.5)	32.5 (15.5)	34.4 (14.5)
Median (min, max)	40 (15, 50)	37.5 (10, 45)	37.5 (10, 50)
**2000Hz**			
Mean (SD)	41.3 (7.5)	52.5 (22.2)	46.9 (16.5)
Median (min, max)	45 (30, 45)	50 (30, 80)	45 (30, 80)
**4000Hz**			

Mean (SD)<br>Median (min, max)	75 (14.8)<br>75 (60, 90)	63.8 (19.3)<br>62.5 (45, 85)	69.4 (17.0)<br>70 (45, 90)
**8000Hz**<br>Mean (SD)<br>Median (min, max)	 80 (23.8)<br>90 (45, 95)	 71.3 (25.0)<br>65 (50, 105)	 75.6 (23.1)<br>80 (45, 105)
**Aetiology, n (%)**<br>Sensorineural<br>Conductive<br>Mixed<br>Other	 3 (75.0)<br>0 (0.0)<br>1 (25.0)<br>0 (0.0)	 4 (100.0)<br>0 (0.0)<br>0 (0.0)<br>0 (0.0)	 7 (87.5)<br>0 (0.0)<br>1 (12.5)<br>0 (0.0)

Table B: International Outcomes Inventory for Hearing Aids (IOI-HA) results for participants, by group and overall (Higher score is better, range 7-35)

	ACE Intervention participants (n=4)	Control participants (n=4)	All participants (n=8)
**Baseline**<br>Mean (SD)<br>Median (min, max)	N=4<br>22.5 (6.6)<br>23 (15, 29)	N=3<br>25.7 (3.1)<br>25 (23, 29)	N=7<br>23.9 (5.3)<br>25 (15, 29)
**Week 5**<br>Mean (SD)<br>Median (min, max)	N=3<br>23.0 (3.6)<br>24 (19, 26)	N=3<br>26.3 (4.7)<br>28 (21, 30)	N=6<br>24.7 (4.2)<br>25 (19, 30)
**3 months post-randomisation**<br>Mean (SD)<br>Median (min, max)	N=3<br>20 (1.7)<br>19 (19, 22)	N=3<br>27.7 (3.5)<br>28 (24, 31)	N=6<br>23.9 (4.9)<br>23 (19, 31)

Table C: Self-Assessment of Communication (SAC) (Lower score is better, range 0 – 100)

	ACE Intervention participants (n=4)	Control Participants (n=4)	All participants (n=8)
**Baseline**<br>Mean (SD)<br>Median (min, max)	N=4<br>65 (21.9)<br>58.8 (47.5, 95)	N=4<br>20 (7.9)<br>21.3 (10, 27.5)	N=8<br>42.5 (28.5)<br>37.5 (10, 95)
**3 months post-randomisation**<br>Mean (SD)<br>Median (min, max)	N=3<br>62.5 (10.9)<br>57.5 (55, 75)	N=3<br>22.5 (4.3)<br>25 (17.5, 25)	N=6<br>42.5 (23.1)<br>40 (17.5, 75)

Table D: International Outcomes Inventory for Alternative Interventions (IOI-AI) (scored 1 – 5, higher score is better)

	ACE intervention participants (n=4)
**Week 5**	N=3
Mean (SD)	2.6 (0.7)
Median (min, max)	3 (1.9, 3.1)
**3 months post-randomisation**	N=2
Mean (SD)	2.6 (0.6)
Median (min, max)	2.6 (2.1, 3)

Table E: International Outcomes Inventory for Hearing Aids: version for Significant Others (IOI-HA-SO) results for significant others, overall (Higher score is better, range 7 - 35)

	All SOs (n=4)
**Baseline**	N=4
Mean (SD)	23.8 (5.3)
Median (min, max)	24 (17, 30)
**Week 5**	N=3
Mean (SD)	25.7 (3.1)
Median (min, max)	25 (23, 29)
**3 months post-randomisation**	N=3
Mean (SD)	24 (4.0)
Median (min, max)	24 (20, 28)

Table F: Significant Other Scale for Hearing Disability (SOS-HEAR) (scored 0 – 4, higher scores indicate greater difficulties experienced by the SO.)

	All SOs (n=4)
**Baseline**	N=4
Mean (SD)	1.0 (0.5)
Median (min, max)	0.9 (0.5, 1.8)
**Week 5**	N=3
Mean (SD)	1.2 (1.0)
Median (min, max)	0.8 (0.5, 2.3)
**3 months post-randomisation**	N=3
Mean (SD)	1.4 (1.0)
Median (min, max)	1.1 (0.6, 2.4)

Table G: Average health care resource use by trial arm at baseline

	ACE				TAU			
	n	Mean (SD)	Min, Max	Missing	n	Mean (SD)	Min, Max	Missing
**GP Visit**	2	0.5 0.71	0 1	2	4	0	0, 0	0
**home**	2	0 (0)	0, 0	2	4	0 (0)	0, 0	0
**Physiotherapist**	2	0 (0)	0, 0	2	4	0 (0)	0, 0	0
**Nurse**	2	0 (0)	0, 0	2	4	0 (0)	0, 0	0
**District/ community nurse**	0	0 (0)	0, 0		4	0 (0)	0, 0	0
**Hearing therapist**	2	1 (1.41)	0, 2	2	3	0.33 (0.58)	0, 1	1
**Speech therapist**	2	0 (0)	0, 0	2	4	0 (0)	0, 0	0
**Counsellor**	2	0 (0)	0, 0	2	4	0 (0)	0, 0	0
**Psychologist**	2	0 (0)	0, 0	2	4	0 (0)	0, 0	0
**Psychiatrist**	2	0 (0)	0, 0	2	4	0 (0)	0, 0	0
**ENT nurse**	2	0 (0)	0, 0	2	4	0.75 (1.5)	0, 3	0
**ENT doctor**	2	0 (0)	0, 0	2	4	0.75 (1.5)	0, 3	0
**Outpatient attendance**	2	1.5 (0.71)	1 2	2	4	1.75 (1.71)	0, 4	0
**A&E attendance**	2	0 (0)	0, 0	2	4	0 (0)	0, 0	0
**Day case**	2	0.5 (0.71)	0, 1	2	4	0 (0)	0, 0	0
**In patient per night**	2	0 (0)	0, 0	2	4	0 (0)	0, 0	0
**Private physiotherapist**	2	0 (0)	0, 0	2	4	0 (0)	0, 0	0
**Private audiologist**	2	0 (0)	0, 0	2	4	0 (0)	0, 0	0
**Private osteopath**	2	0 (0)	0, 0	2	4	0 (0)	0, 0	0
**Private counsellor**	2	0 (0)	0, 0	2	4	0 (0)	0, 0	0
**Private acupuncturist**	2	0 (0)	0, 0	2	4	0 (0)	0, 0	0
**Private chiropractitioner**	2	0 (0)	0, 0	2	4	0 (0)	0, 0	0
**Private day case**	2	0 (0)	0, 0	2	4	0 (0)	0, 0	0
**Private in patient per night**	2	0 (0)	0, 0	2	4	0 (0)	0, 0	0

Table H: Average health care resource use by trial arm at 3 months post-randomisation

	ACE				TAU			
	**n**	**Mean (SD)**	**Min, Max**	**Missing**	**n**	**Mean (SD)**	**Min, Max**	**Missing**
**GP Visit**	1	0 (0)	0, 0	3	3	0 (0)	0, 0	1
**home**	1	0 (0)	0, 0	3	3	0 (0)	0, 0	1
**Physiotherapist**	1	0 (0)	0, 0	3	3	0 (0)	0, 0	1
**Nurse**	0	-	-	4	3	0 (0)	0, 0	1
**District/ community nurse**	1	0 (0)	0, 0	3	3	0 (0)	0, 0	1
**Hearing therapist**	2	0.5 (0.71)	0, 1	2	3	0 (0)	0, 0	1
**Speech therapist**	1	0 (0)	0, 0	3	3	0 (0)	0, 0	1
**Counsellor**	1	0 (0)	0, 0	3	3	0 (0)	0, 0	1
**Psychologist**	1	0 (0)	0, 0	3	3	0 (0)	0, 0	1
**Psychiatrist**	1	0 (0)	0, 0	3	3	0 (0)	0, 0	1
**ENT nurse**	1	1 (-)	1, 1	3	3	0 (0)	0, 0	1
**ENT doctor**	1	0 (0)	0, 0	3	3	0 (0)	0, 0	1
**Outpatient attendance**	1	1 (-)	1, 1	3	3	0 (0)	0, 0	1
**A&E attendance**	1	0 (0)	0, 0	3	3	0 (0)	0, 0	1
**Day case**	1	0 (0)	0, 0	3	3	0 (0)	0, 0	1
**In patient per night**	1	0 (0)	0, 0	3	3	0 (0)	0, 0	1
**Private physiotherapist**	1	0 (0)	0, 0	3	3	0 (0)	0, 0	1
**Private audiologist**	1	2 (-)	2, 2	3	3	0 (0)	0, 0	1
**Private osteopath**	1	0 (0)	0, 0	3	3	0 (0)	0, 0	1
**Private counsellor**	1	0 (0)	0, 0	3	3	0 (0)	0, 0	1
**Private acupuncturist**	1	0 (0)	0, 0	3	3	0 (0)	0, 0	1
**Private chiropractitioner**	1	0 (0)	0, 0	3	3	0 (0)	0, 0	1
**Private day case**	1	0 (0)	0, 0	3	3	0 (0)	0, 0	1
**Private in patient per night**	1	0 (0)	0, 0	3	3	0 (0)	0, 0	1
