# Supplementary material for: Randomised controlled feasibility trial of an active communication education programme plus hearing aid provision versus hearing aid provision alone (ACE To HEAR)
Source: BMJ Open. 2021 Apr 7;11(4):e043364. doi: 10.1136/bmjopen-2020-043364 (PMC8031014; doi:10.1136/bmjopen-2020-043364)
Supplement: Author's manuscript [file bmjopen-2020-043364.draft_revisions.pdf]

BMJ Open

BMJ Open is committed to open peer review. As part of this commitment we make the peer review history of every article we publish publicly available.

When an article is published we post the peer reviewers' comments and the authors' responses online. We also post the versions of the paper that were used during peer review. These are the versions that the peer review comments apply to.

The versions of the paper that follow are the versions that were submitted during the peer review process. They are not the versions of record or the final published versions. They should not be cited or distributed as the published version of this manuscript.

BMJ Open is an open access journal and the full, final, typeset and author-corrected version of record of the manuscript is available on our site with no access controls, subscription charges or pay-per-view fees (http://bmjopen.bmj.com).

If you have any questions on BMJ Open's open peer review process please email info.bmjopen@bmj.com

BMJ Open

A randomised controlled feasibility trial of an Active Communication Education programme plus hearing aid provision versus hearing aid provision alone (ACE To HEAR)

Journal:	*BMJ Open*
Manuscript ID	bmjopen-2020-043364
Article Type:	Original research
Date Submitted by the Author:	31-Jul-2020
Complete List of Authors:	Watson, Judith; University of York, Health Sciences; University of York<br>Coleman, Elizabeth; York Trials Unit, Health Sciences<br>Jackson, Cath; University of York, York Trials Unit<br>Bell, Kerry; University of York, Health Sciences<br>Maynard, Christina; Univerity of Leeds, Leeds Institute of Cardiovascular & Metabolic Medicine<br>Hickson, Louise; The University of Queensland, School of Health and Rehabilitation Sciences<br>Forster, Anne; University of Leeds, Academic Unit of Elderly Care and Rehabilitat<br>Fairhurst, Caroline; University of York, Health Sciences<br>Hewitt, Catherine; The University of York, Health Sciences<br>Gardner, Rob; Bradford Royal Infirmary, Audiology Department<br>Iley, Kate; York Teaching Hospital NHS Foundation Trust, Audiology<br>Gailey, Lorraine; Hearing Link<br>Thyer, Nicholas; Univerity of Leeds, Leeds Institute of Cardiovascular & Metabolic Medicine
Keywords:	STATISTICS & RESEARCH METHODS, QUALITATIVE RESEARCH, Adult otolaryngology < OTOLARYNGOLOGY

SCHOLARONE™
Manuscripts

I, the Submitting Author has the right to grant and does grant on behalf of all authors of the Work (as defined in the below author licence), an exclusive licence and/or a non-exclusive licence for contributions from authors who are: i) UK Crown employees; ii) where BMJ has agreed a CC-BY licence shall apply, and/or iii) in accordance with the terms applicable for US Federal Government officers or employees acting as part of their official duties; on a worldwide, perpetual, irrevocable, royalty-free basis to BMJ Publishing Group Ltd ("BMJ") its licensees and where the relevant Journal is co-owned by BMJ to the co-owners of the Journal, to publish the Work in this journal and any other BMJ products and to exploit all rights, as set out in our licence.

The Submitting Author accepts and understands that any supply made under these terms is made by BMJ to the Submitting Author unless you are acting as an employee on behalf of your employer or a postgraduate student of an affiliated institution which is paying any applicable article publishing charge ("APC") for Open Access articles. Where the Submitting Author wishes to make the Work available on an Open Access basis (and intends to pay the relevant APC), the terms of reuse of such Open Access shall be governed by a Creative Commons licence – details of these licences and which Creative Commons licence will apply to this Work are set out in our licence referred to above.

Other than as permitted in any relevant BMJ Author's Self Archiving Policies, I confirm this Work has not been accepted for publication elsewhere, is not being considered for publication elsewhere and does not duplicate material already published. I confirm all authors consent to publication of this Work and authorise the granting of this licence.

For peer review only

A randomised controlled feasibility trial of an Active Communication Education programme plus hearing aid provision versus hearing aid provision alone (ACE To HEAR)

Judith Watson- York Trials Unit, Department of Health Sciences, University of York, ARRC Building, York, UK (Orcid iD 0000-0003-0694-3854)

Elizabeth Coleman - York Trials Unit, Department of Health Sciences, University of York, ARRC Building, York, UK (Orcid iD – 0000-0003-4210-1865)

Cath Jackson - Valid Research Ltd, Wetherby, UK (Orcid iD 0000-0003-3181-7091, https://uk.linkedin.com/in/cath-jackson-valid-research)

Kerry Bell- York Trials Unit, Department of Health Sciences, University of York, ARRC Building, York, UK (Orcid iD 0000-0001-5124-138X)

Christina Maynard - Leeds Institute of Cardiovascular and Metabolic Medicine (LICAMM,) University of Leeds, Leeds, UK and Bramhope Medical Centre, Leeds, UK

Louise Hickson - School of Health and Rehabilitation Sciences, University of Queensland, Brisbane, Queensland 4072, Australia (Orcid Id: 0000-0001-6832-4173) Twitter: @Louise_Hickson LinkedIn: https://www.linkedin.com/in/louise-hickson-ba273640/

Anne Forster - Leeds Institute of Health Sciences (LIHS), University of Leeds, Leeds, UK

Caroline Fairhurst- York Trials Unit, Department of Health Sciences, University of York, ARRC Building, York, UK (Orcid iD 0000-0003-0547-462X)

Catherine Hewitt- York Trials Unit, Department of Health Sciences, University of York, ARRC Building, York, UK (Orcid iD 0000-0002-0415-3536)

Rob Gardner -Audiology Department, Bradford Royal Infirmary, Bradford, UK

Kate Iley - Audiology Department, York Hospital, York, UK

For peer review only - http://bmjopen.bmj.com/site/about/guidelines.xhtml

Lorraine Gailey- Hearing Link, Eastbourne, UK

Nicholas J Thyer – Leeds Institute of Cardiovascular and Metabolic Medicine (LICAMM,) University of Leeds, Leeds, UK (Orcid iD 0000-0002-0025-783X)

Corresponding author: Dr Judith Watson: jude.watson@york.ac.uk

Total word count of main text= 5144

For peer review only

For peer review only - http://bmjopen.bmj.com/site/about/guidelines.xhtml

Abstract:

Objective: To establish the acceptability and feasibility of delivering the Active Communication Education (ACE) programme to increase quality-of-life through improving communication and hearing aid use in the UK National Health Service.

Design: Randomised controlled, open feasibility trial with embedded economic and process evaluations.

Setting: Audiology Departments in two hospitals in two UK cities.

Participants: Twelve hearing aid users aged 18 years or over who reported moderate or less than moderate benefit from their new hearing aid.

Interventions: Consenting participants (along with a significant other) were to be randomised by a remote, centralised randomisation service in groups to Active Communication Education (ACE) plus treatment-as-usual (intervention group) or treatment-as-usual only (control group).

Primary outcome measures: The primary outcomes were related to feasibility: recruitment, retention, treatment adherence and acceptability to participants, and fidelity of treatment delivery.

Secondary outcome measures: International Outcomes Inventory for Hearing Aids, Self-Assessment of Communication, EQ-5D-5L and Short-Form 36. Blinding of the participants and facilitator was not possible.

Results: Twelve hearing aid users and six significant others consented to take part. Eight hearing aid users were randomised: four to the intervention group; and four to treatment-as-usual only. Four significant others participated alongside the randomised participants. Recruitment to the study was very low and centres only screened 466 hearing aid users over the 15-month recruitment period, compared with the approximately 3,500 anticipated. Only one ACE group and

For peer review only - http://bmjopen.bmj.com/site/about/guidelines.xhtml

one control group were formed. ACE could be delivered and appeared acceptable to participants. We were unable to robustly assess attrition and attendance rates due to the low sample size.

Conclusions: Whilst ACE appeared acceptable to hearing aid users and feasible to deliver, it was not feasible to identify and recruit participants struggling with their hearing aids at the 3-month post-hearing aid fitting point.

Trial registration: ISRCTN28090877

Word count: 293

Keywords: Active Communication Education; Audiology; Feasibility; Hearing Aid; Randomised Controlled Trial; Quality of Life

Strengths and limitations of this study:

- This is the first study to explore the potential of using ACE as a tool to improve communication and hearing aid benefit for struggling adult hearing aid users and their significant others.
- This is the first study to identify and evaluate the processes needed to deliver an RCT of treatment-as-usual plus ACE versus treatment-as-usual alone within audiology clinics.
- Low recruitment meant some of the trial objectives were not robustly assessed.

For peer review only

For peer review only - http://bmjopen.bmj.com/site/about/guidelines.xhtml

INTRODUCTION

Age-related hearing impairment is reported as the third most common chronic condition affecting approximately 328 million (91%) middle and older aged adults worldwide[1] and 10 million adults in the United Kingdom (UK) [2,3] with the majority having mild or worse hearing impairment, which progressively deteriorates with age.[4] Hearing loss often impacts on quality-of-life and general well-being [5,6,7] such as increased occurrences of depression, [8] social isolation, [7] poor social interactions, [9,10,11] and cognitive dysfunction,[12] as well as increased risk of developing dementia.[13] In addition the impact on normally-hearing significant others (SOs) living with hearing-impaired people, is often overlooked where similar problems are reported.[9,14,15,16,17]

In spite of robust evidence that hearing aid use reduces the negative impact of age-related hearing impairment on quality-of-life,[5,18,19,20] it is estimated that up to 30% of UK adult hearing aid owners do not use them regularly or at all.[3,21,22,23] The reasons behind this are complex. [24] Numerous barriers and facilitators to successful hearing aid use have been identified as being related to expectations of benefit and meaningful participation in everyday life. [25,26]

The Active Communication Education (ACE) programme [27] trains participants to develop solutions to specific difficult communication scenarios that commonly lead to their avoidance of, or reduced participation in, daily activities. Three small studies have demonstrated benefits in improving communication function and hearing related quality-of-life when evaluated as an *alternative* to hearing aid fitting.[28,29,30] However, the effect of ACE on improving hearing aid benefit when delivered *in addition* to hearing aid fitting has not been evaluated. The ACE To HEAR study (**A**ctive **C**ommunication **E**ducation **To** improve **HEAR**ing) aimed to assess whether a large randomised controlled trial (RCT) to evaluate the effectiveness and cost-effectiveness of ACE plus

For peer review only - http://bmjopen.bmj.com/site/about/guidelines.xhtml

treatment-as-usual (TAU) versus TAU alone, was feasible within UK National Health Service (NHS) Audiology Departments.

DESIGN AND METHODS

The methods are summarised below and published in more detail elsewhere.[31] This was an open feasibility RCT with embedded economic and process evaluations. The required regulatory approvals were in place prior to study commencement.

Setting

The participating centres were York Hospital, York Teaching Hospitals NHS Foundation Trust, and Bradford Royal Infirmary, Bradford Teaching Hospitals NHS Foundation Trust.

Intervention

TAU was defined as a referral from a GP to audiology services to treat permanent hearing loss. It comprised of up to two appointments for hearing aid fitting and a third face-to-face or telephone follow-up appointment. The ACE to HEAR intervention consisted of five group sessions delivered as described in the published ACE manual [27] by a trained audiologist facilitator to groups of 5-7 hearing aid users and their SOs, where possible.

Study objectives

The study objectives were established around evaluating the delivery of the ACE intervention and the feasibility of trial delivery to assess:

- trial up-take rates, eligibility, and acceptability of clinic location;
- recruitment rates, the randomisation process and time to accrue ACE groups;

For peer review only - http://bmjopen.bmj.com/site/about/guidelines.xhtml

- ACE attendance and retention amongst participants randomised to the intervention group;
- acceptability of ACE with participants, SOs and audiologists;
- capability, capacity and willingness of audiology departments to support delivery of ACE within existing services;
- intervention fidelity of delivering ACE;
- the acceptability of study processes to participants, SOs and audiologists;
- patient reported outcome measures (PROMs) including piloting a bespoke resource use questionnaire and estimate parameters to be used when designing full-scale RCT.

Sample size and randomisation

Since this was a feasibility trial, no formal power calculation was conducted. We aimed to recruit 88 hearing aid (HA) users aged 18 years or over receiving TAU in the two participating centres, along with their SOs. The full eligibility criteria can be found in the published protocol.[31] The study sites estimated that there would be approximately 400 new hearing aids fitted across the study centres each month. Assuming 10% eligibility, 35% did not attend (DNA) and 30% consent rate, around 350 cases per month across the two centres would have needed to be screened to recruit the required 88 HA users in 12 months.

In the initial months of the trial (April to August 2017), eligibility criteria had included Q1 and Q2 from the International Outcomes Inventory for Hearing Aids (IOI-HA). [32] Q1 required self-reported HA use of less than three hours a day and Q2 required *less than moderate* benefit for eligibility. However, early scrutiny of the Q1 recruitment data showed evidence of over reported hearing aid use, including a variable presence of confirmatory HA log data and it was therefore removed as a criterion. Q2 was also reviewed and considered too stringent; eligibility was changed to include

For peer review only - http://bmjopen.bmj.com/site/about/guidelines.xhtml

moderate as well as *less than moderate benefit* as these HA users were also considered to be struggling. These changes were implemented as of 29th August 2017.

Eligible, consenting HA users from the same study site who had completed baseline assessments were to be randomised 1:1 by a remote, centralised randomisation service (provided by York Trials Unit) in batches of 10–14 using block randomisation in a single large block per batch. This was to allow groups of 5-7 to be formed for the ACE intervention sessions. The target was to randomise participants within three weeks of consent.

Measurements

Data collected from participants at baseline via self-completed questionnaires were: IOI-HA;[32] Self-Assessment of Communication (SAC);[33] EQ-5D-5L;[34,35] and Short-Form 36 (SF-36)[36], resource use and demographics. SOs completed baseline questionnaires collecting: demographics; IOI-HA: version for Significant Others (IOI-HA-SO);[37] and Significant Other Scale for Hearing Disability (SOS-HEAR).[14]

Those in the ACE group also completed a questionnaire at the end of ACE session five. Measures were IOI-HA and the International Outcomes Inventory for Alternative Interventions (IOI-AI).[38] Their SOs completed IOI-HA-SO, SOS-HEAR, and the International Outcome Inventory for Alternative Interventions: version for Significant Others (IOI-AI-SO).[37]

The TAU participants were mailed questionnaires containing the IOI-HA at the equivalent time point; with their SOs completed IOI-HA-SO and SOS-HEAR. Participants and their SOs were asked additional questions regarding their thoughts on the programme (ACE arm only) and being

For peer review only - http://bmjopen.bmj.com/site/about/guidelines.xhtml

approached to take part in the study (both arms). The facilitator collected data during all five ACE sessions in relation to participants' attendance at sessions, SO attendance and their relationship to participant.

At three months post-randomisation both groups (ACE and TAU) and their SOs completed the same measures as at baseline, and those in the ACE group and their SOs also completed the IOI-AI and associated IOI-AI-SO.

The feasibility and the potential for a future large-scale study was to be determined by a set of stop: go criteria (Table 1).

Table 1: Stop: go criteria

Criteria	Measure
Seventy per cent of recruitment targets attained for all research components	Numbers referred; numbers struggling with their hearing aid; number of exclusions.
Study consent/retention rates and proposed sample sizes, indicate delivery of the full RCT is plausible within a 5-year study period	Numbers screening; numbers consenting; numbers who declined to participate and reasons for declining; numbers withdrawing.
Ninety per cent of ACE groups of five to seven consented participants formed within the intervention window with participants attending three of five sessions	Time taken to recruit and logistics of recruiting an optimally sized and located ACE group; time ACE started after randomisation (ACE intervention window). Number of consented participants who failed to attend ACE sessions; number who missed ACE intervention window (i.e. unable to attend an ACE group within 1-3 weeks after randomisation).
Economic, acceptability, outcome measure and fidelity evaluation data successfully collect	Amount of missing data, although measures with over 10% missing data may be modified/replaced prior to the main trial.

For peer review only - http://bmjopen.bmj.com/site/about/guidelines.xhtml

Participants, SOs and audiologists evaluate acceptability of the ACE and RCT positively	Number given an appointment for an ACE group session; number of consented participants who failed to attend ACE sessions; Facilitator's adherence to the ACE protocol (fidelity); participant and SO thoughts regarding the study (interviews)

Statistical analysis

A single analysis was conducted at the end of the trial and undertaken in Stata v15. Baseline and outcome data for HA users are summarised overall and by randomised group, and are reported as mean, standard deviation (SD), median, minimum and maximum for continuous data, and counts and percentages for categorical data. The amount of missing data for each variable is also reported. The recruitment rate is summarised by month, overall, and by site. Attendance at the ACE sessions by participants and their SOs are detailed.

Questionnaire return rates at each time point are presented overall and by group. It was planned that SDs would be presented for PROMs with 80% confidence intervals to inform future sample size calculations. An analysis to investigate sensitivity of the measures to change, by calculating the standardised response mean, was also planned.

The number of participants withdrawing from the ACE intervention and/or the trial and any reasons for withdrawal are summarised.

Economic analysis and quality of life data

The present trial aimed to determine the feasibility of conducting an economic evaluation of the ACE to HEAR intervention taking a broad perspective accounting not only for NHS costs but also

For peer review only - http://bmjopen.bmj.com/site/about/guidelines.xhtml

those observed by HA users. In the UK, NICE advocates the use of the EQ-5D for measuring quality-of-life; however, the EQ-5D is not always the most sensitive tool for particular populations for whom the majority of its dimensions may not apply.[39] In a United States analysis of a similar population, the SF-36 was used to generate QALYs rather than the EQ-5D, with the SF-36 showing a reduction in the cost per QALY in favour of the intervention.[28] Given the UK preference for the EQ-5D, and the fact that it is shorter, thus less onerous to complete, participants in the feasibility trial were asked to complete both instruments to allow for comparison. Completion rates and mean scores for both instruments are summarised.

A bespoke resource use questionnaire was also designed to estimate the potential resource implications of the intervention versus usual care. Completion rates and mean resource use by item are summarised.

Process evaluation

The number of ACE sessions delivered was recorded and, to assess fidelity of delivering ACE, the facilitator completed a self-monitoring form, scoring adherence to the ACE manual for each session.

Following delivery of all the sessions, the facilitator was interviewed to explore the training and implementation process and their experiences of delivering ACE including barriers/facilitators to adhering to the ACE protocol. In addition, three audiologists were interviewed, exploring the capability, capacity and willingness of their audiology departments to support the ACE study within their existing services. The acceptability of study processes were also explored.

For peer review only - http://bmjopen.bmj.com/site/about/guidelines.xhtml

Three participants in the ACE intervention group, plus one SO, were interviewed after the completion of the ACE sessions. The semi-structured interviews explored the acceptability of the ACE (e.g. venue, timing, content), its perceived impact (reflecting on HA outcomes) as well as exploration of participant's views on study processes (e.g. recruitment, outcome measures and timing).

Interviews were audio-recorded, transcribed verbatim and analysed (with NVivo-11), using the Framework approach,[40] and then interpreted in by team members.

RESULTS

Recruitment

The flow of participants through the trial is shown in a CONSORT diagram (Figure 1). Screening and recruitment occurred from 01st April 2017 to the end of June 2018; 466 HA users were screened whilst attending a follow-up HA fitting appointment at Bradford (n=275) or contacted by telephone 3-months post-fitting at York (n=191). Of these, 86 (18.5%) were eligible but 74 declined to participate. Twelve participants were recruited into the trial (14.0% of those eligible); one from Bradford and eleven from York (Figure A in supplementary material). Reasons for ineligibility (n=380) and non-consent (n=74) can be found in Figure 1. The most common reason was that the HA helped too much (81.8% of n=380 excluded). Six SOs also consented to take part.

It was found that 22.6% of HA users who were screened (n=380) were eligible (based on the applicable screening criteria at the time of screening; additional participants were identified as 'would have been eligible' when the inclusion criteria were changed); however, on average only 33

For peer review only - http://bmjopen.bmj.com/site/about/guidelines.xhtml

HA users were screened each month, and this was confounded by the 13.9% consent rate (% of the 86 eligible, 8.9% when including those who would have been eligible under the new criteria).

Randomisation

Randomisation was intended to be performed in batches of 10-14 participants. However, due to slow recruitment, the decision was made in January 2018 to randomise the participants recruited from York thus far as a smaller block to allow at least one ACE group to be run. The randomisation was undertaken as a block of eight, four participants to each group. Additionally, four SOs were also recruited, and allocated to their respective participant's group – three to TAU and one to the intervention group. There were insufficient numbers of participants to randomise the four subsequently recruited participants, and the two SOs.

The average time from screening to randomisation was 19.5 weeks (range 10.6 to 33.6 weeks) and the target three-week window for randomisation post-consent was missed for all participants. If the group had been randomised immediately after the eighth participant was consented (randomisation was delayed in the hope of a larger group), then the average time would have been 8.9 weeks, with 37.5% meeting the three-week target. The first ACE session was conducted 11 days after randomisation.

Participants

The baseline characteristics of randomised participants and their SOs can be found in Table 2. As there was only one SO in the intervention group, data are only presented overall to prevent disclosure.

For peer review only - http://bmjopen.bmj.com/site/about/guidelines.xhtml

Table A in the supplementary material shows the screening data on use and benefit of HA, threshold data and hearing loss aetiology for the randomised participants by group and overall.

Table 2: Baseline characteristics for the randomised participants, by group and overall and overall for the SOs

	ACE Intervention participants (n=4)	TAU participants (n=4)	All participants (n=8)	All SOs (n=4)
**Age, years**				
Mean (SD)	73.0 (7.8)	73.3 (6.0)	73.1 (6.4)	73.6 (7.3)
Median (min, max)	72.9 (64.1, 82.1)	73.9 (65.9, 79.5)	73.6 (64.1, 82.1)	76.6 (62.9, 78.3)
**Gender, n (%)**				
Male	3 (75.0)	2 (50.0)	5 (62.5)	2 (50.0)
Female	1 (25.0)	2 (50.0)	3 (37.5)	2 (50.0)
**Length of time concerned about hearing, years**		3.5 (1.9)	12.4 (10.8)	N/A
Mean (SD)	21.3 (7.5)	4 (1, 5)	10 (1, 30)	
Median (min, max)	20 (15, 30)			
**Ethnicity, n (%)**				
White British	4 (100.0)	4 (100.0)	8 (100.0)	4 (100.0)
Other	0 (0.0)	0 (0.0)	0 (0.0)	0 (0.0)
**Marital Status, n (%)**				
Married/Civil partner	2 (50.0)	3 (75.0)	5 (62.5)	4 (100.0)
Separated	0 (0.0)	0 (0.0)	0 (0.0)	0 (0.0)
Divorced	1 (25.0)	1 (25.0)	2 (25.0)	0 (0.0)
Missing	1 (25.0)	0 (0.0)	1 (12.5)	0 (0.0)
**Living arrangements, n (%)**				
Owned outright	3 (75.0)	4 (100.0)	7 (87.0)	4 (100.0)
Owned with mortgage	0 (0.0)	0 (0.0)	0 (0.0)	0 (0.0)
Rented	0 (0.0)	0 (0.0)	0 (0.0)	0 (0.0)
Privately rented	1 (25.0)	0 (0.0)	1 (12.5)	0 (0.0)
**Main activity, n (%)**				
Full time employment	0 (0.0)	0 (0.0)	0 (0.0)	0 (0.0)
Part time	0 (0.0)	0 (0.0)	0 (0.0)	0 (0.0)
Self-employed	0 (0.0)	0 (0.0)	0 (0.0)	0 (0.0)

For peer review only - http://bmjopen.bmj.com/site/about/guidelines.xhtml

Unable to work due to health	1 (25.0)	0 (0.0)	1 (12.5)	0 (0.0)
Retired	2 (50.0)	4 (100.0)	6 (75.0)	4 (100.0)
Student	0 (0.0)	0 (0.0)	0 (0.0)	0 (0.0)
Retired & self-employed	1 (25.0)	0 (0.0)	1 (12.5)	0 (0.0)
**Qualifications, n (%)**				
No formal qualifications	2 (50.0)	1 (25.0)	3 (37.5)	0 (0.0)
GCSEs	1 (25.0)	1 (25.0)	2 (25.0)	1 (25.0)
A/AS Levels	0 (0.0)	0 (0.0)	0 (0.0)	0 (0.0)
Higher education	0 (0.0)	1 (25.0)	1 (12.5)	2 (50.0)
Further higher education	0 (0.0)	1 (25.0)	1 (12.5)	0 (0.0)
Vocational	0 (0.0)	0 (0.0)	0 (0.0)	0 (0.0)
Other	1 (25.0)	0 (0.0)	1 (12.5)	1 (12.5)

Participants had mild to moderately severe high frequency sensorineural hearing loss, except for one who reported an additional conductive element to their hearing loss. Five participants reported using their HA for less than 4 hours a day and three for more than 8 hours a day. All participants reported less than moderate levels of benefit. Those randomised to the ACE intervention reported that they had been concerned about their hearing for markedly more years than those allocated to TAU.

Intervention attendance

Of the four York participants who were randomised to receive the intervention, one attended all five sessions, one attended four sessions, one three sessions, and one participant did not attend any of the five sessions. This equates to an average attendance rate of 60%. Of these four participants, only one had a SO who wished to participate, and they attended all the sessions that their associated participant attended.

For peer review only - http://bmjopen.bmj.com/site/about/guidelines.xhtml

Follow-up and standardised measures

At the end of the sessions (approximately week 5), 7/8 participants (87.5%) returned the postal questionnaire (ACE 4/4 (100%); TAU 3/4 (75%)). In addition, 3/4 (75%) SOs returned their questionnaires (ACE 1/1 (100%); TAU 2/3 (67%)).

At 3-months post-randomisation, the retention rate was 75%; three out of four participants from each group returned the questionnaires. Three of the four (75%) SOs returned their 3-month post-randomisation questionnaires. No participants withdrew in this study.

The results from Tables B – D (supplementary material), completed by the participants at both baseline and at least one of the follow-up time points, show that the measures used were well completed. The average IOI-HA score at all three time points was in the mid-to-high range, indicating average hearing outcomes; there was no obvious change in this result in either group over time. There does appear to be imbalance in the SAC between TAU and ACE participants, with those in the control group rating themselves as being worse at communicating. However, as with the IOI-HA there appears to be no change in the average score across the follow-up period.

The outcome measures for the SOs were equally well completed (Tables E & F: supplementary material). Since only one SO completed the IOI-AI-SO, the results are not presented.

Out of all of the returned questionnaires (n=8, 6 and 6 for participants and n=4, 3 and 3 for SOs at baseline, W5 and M6 respectively) there was only one instance where a PROM was not completed sufficiently to allow for scoring (IOI-AI for an ACE participant at M6).

For peer review only - http://bmjopen.bmj.com/site/about/guidelines.xhtml

The 80% confidence intervals for SDs for PROMs were not calculated, and the planned analysis to investigate the sensitivity of the measures to change was not conducted as it was considered they would not produce reliable results due to the low sample size.

Health Economics

Of the eight participants at baseline, all completed the EQ-5D-5L in full, compared to six full completions (75%) and two partial completions for the SF-36 (25%). At 3-months post-randomisation, two participants (one from each group) did not complete the questionnaire; all remaining participants completed both the EQ-5D-5L and SF-36 in full. At baseline, participants allocated to the intervention group had a slightly higher mean score compared to participants in the TAU group (mean difference 0.057, 95% CI -0.691 to 0.805) (Table 3). This difference was reversed at 3 months with participants allocated to the TAU group having a slightly higher mean score (mean difference -0.186, 95% CI -0.878 to 0.506).

Table 3: EQ-5D-5L mean (SD) scores and unadjusted mean difference (95% CI) at baseline and 3 months post-randomisation follow-up

Follow-up	ACE Mean (SD)	TAU Mean (SD)	Difference (ACE-TAU) (95% CI)
Baseline	0.670 (0.333)	0.613 (0.512)	0.057 (-0.691; 0.805)
3 Months post-randomisation	0.686 (0.411)	0.871 (0.133)	-0.186 (-0.878; 0.506)

These findings were also reflected in the results of the SF-36 (Table 4). At baseline, participants allocated to the intervention group scored on average higher on dimensions of physical role, general health, social function, emotional role and mental health as well as on the overall physical and

For peer review only - http://bmjopen.bmj.com/site/about/guidelines.xhtml

mental components. At the 3-month post-randomisation follow-up, participants allocated to the intervention group only scored higher on the measure of vitality.

Table 4: SF-36 mean (SD) scores and unadjusted mean difference (95% CI) at baseline and 3 months post-randomisation follow-up

SF-36 Score		ACE Mean (SD)	TAU Mean (SD)	Unadjusted difference (95% CI)
**Baseline**				
	Physical functioning	61.67 (28.77)	66.25 (44.60)	-4.58 (-69.52; 60.36)
	Physical role	54.69 (40.63)	48.44 (38.32)	6.25 (-62.07; 74.57)
	Bodily pain	59 (32.19)	65.33 (46.88)	-6.33 (-82.39; 69.72)
	General health	61.5 (36.30)	49.75 (24.31)	11.75 (-41.70; 65.20)
	Vitality	45.31 (22.46)	52.08 (15.73)	-6.77 (-46.12; 32.58)
	Social functioning	68.75 (33.07)	58.33 (50.52)	10.42 (-69.99; 90.82)
	Emotional role	77.08 (26.68)	77.08 (15.77)	0 (-37.92; 37.92)
	Mental health	68.75 (23.94)	63.33 (12.58)	5.42 (-34.20; 45.03)
	Overall physical component	41.11 (14.69)	39.44 (24.22)	1.67 (-35.80; 39.14)
	Overall mental component	46.87 (10.77)	45.86 (2.31)	1.01 (-15.56; 17.57)
**3 Months post-randomisation**				
	Physical functioning	45 (39.05)	83.33 (10.41)	-38.33 (-103.12; 26.45)
	Physical role	41.67 (38.19)	68.75 (22.53)	-27.08 (-98.16; 43.99)
	Bodily pain	66.67 (57.74)	70 (6.93)	-3.33 (-96.55; 89.89)
	General health	58 (41.87)	58.67 (15.28)	-0.67 (-72.12; 70.78)
	Vitality	54.17 (36.62)	52.08 (3.61)	2.08 (-56.90; 61.07)
	Social functioning	62.5 (45.07)	87.5 (21.65)	-25 (-105.15; 55.15)
	Emotional role	75 (25.0)	88.89 (19.25)	-13.89 (-64.46; 36.68)
	Mental health	78.33 (20.21)	78.33 (16.07)	0 (-41.3888; 41.3888)
	Overall physical component	36.10 (21.08)	45.84 (7.134)	-9.74 (-45.41; 25.93)

For peer review only - http://bmjopen.bmj.com/site/about/guidelines.xhtml

Overall mental component	51.33 (10.23)	51.67 (11.97)	-0.34 (-25.58; 24.90)

The resource use questionnaire was not well completed, with one full completion (12.5%) and five partial completions (62.5%) at baseline and six partial completions only at 3 months post-randomisation (75%). There was no difference in completion by trial group. Overall, very low levels of resource use were reported across both groups with most resource use centred on hearing health care professionals (Tables G and H: supplementary material).

Appropriate unit costs to be applied for each resource use type were identified through local costings and national databases including PSSRU [41] and NHS reference costs.[42]

Adverse events

None were recorded.

Process evaluation

Five ACE sessions were delivered. At the first session, (1) introduction to ACE and communication needs analysis, the group decided which modules they wanted to use, since each addressed a different aspect of communication. They chose: conversation in background noise (session 2); conversation around the house (session 3); communication with difficult speakers (session 4); and public-address systems (session 5). Three HA users and one significant other attended.

Facilitator views and fidelity

For peer review only - http://bmjopen.bmj.com/site/about/guidelines.xhtml

Overall, the facilitator considered the ACE programme to have had a positive impact because some of the content was new knowledge for participants, and a few had reported implementing some communication strategies with success.

"The information I was able to give I think was really positive and helped them a lot. I think having spoken to them the following week they'd been able to implement some of them (communication strategies) and found those quite useful. Particularly things like watching the TV at home, that they could put the subtitles on or could change the sound settings or that kind of thing. I think they all felt a bit more empowered by the end of it, that they were able to admit that they had a hearing loss and admit that things weren't going to be perfect in every situation."

Key challenges to its delivery were low numbers of participants which limited discussion, some reluctance amongst participants to "bother others" to help with communication, and participants wanting to discuss hearing aid technology and their frustrations with audiology services (instead of communication). As the sessions progressed, the facilitator became more flexible in delivering the programme, ensuring that these other topics were covered. This was reflected in the fidelity scores which changed from "adherence to the protocol" all the time (session 1), most of the time (sessions 2 and 3) and sometimes (sessions 4 and 5).

Participants' views

In reflecting on the ACE programme, two participants and one SO gave several examples of how it was helpful. They had tried out different strategies: sitting with their back to the wall, adjusting settings on their TV, using subtitles and checking if the loop system is switched on when visiting theatres. A key change was increased confidence in speaking out when they cannot hear and asking others to help with this, for example; facing them when in conversation, requesting people to repeat themselves rather than speak more loudly and closing a window to reduce background noise.

For peer review only - http://bmjopen.bmj.com/site/about/guidelines.xhtml

"It also brought it home to me how important it is to tell other people that you've got a hearing problem, because you're reluctant to do it when you're first deaf. And so you muddle along and you say "yes, yes", and then sometimes you know that they're getting annoyed so then you pretend to hear what was said and of course you laugh in the wrong place or you can't join in the jokes. I always miss punchlines. So, I learned that you must say to people "I'm sorry, I can't hear, can I sit here? Can we do this? Can we turn that off?"

That said, there was still some reluctance to ask others for help, with participants favouring strategies that they could do themselves. One participant said he felt the training "wasn't right for me" and that the sessions had not changed anything for him.

Audiologists' views

The audiologists said they had been able to deliver the study within their departments; adapting their routine follow-up process to include the ACE screening questionnaire. However, the study was more time consuming than anticipated and requiring extra support from the university team to help answer questions from HA users was mentioned in one department. Suggested reasons for the failure to recruit were: too strict eligibility criteria (which were changed); a reluctance of older people to participate in research; communication barriers are already discussed in routine appointments; and many HA users nowadays are younger and want to focus on the benefits that technology afford. Ideas for improving recruitment were: to recruit people at their initial fitting, or people who are long term HA users; offer more days and locations for follow-up appointments so more people attend; present ACE as a support group rather than a research study; and send flyers out or invite potential participants in for a coffee morning to discuss the study.

For peer review only - http://bmjopen.bmj.com/site/about/guidelines.xhtml

Finally, in discussing trial processes, the facilitator and participants observed it had taken a long time to set up the first ACE group and that the information needed to be clearer that the focus of the programme was communication. Participants and audiologists mentioned the length and complexity of the ACE questionnaires. It was difficult to deliver these over the telephone with some elderly HA users.

DISCUSSION

This is the first study to explore the potential of ACE as a tool to improve communication and hearing aid benefit for struggling adult hearing aid users and their significant others. It is also the first study to identify and evaluate the processes needed to deliver an RCT of ACE within the general practitioner direct referral pathway for new NHS hearing aid users in the UK (TAU). The key findings from this feasibility study are: (1) there is some (limited data) to suggest the intervention is potentially acceptable and feasible to HA users, SOs and audiologists; (2) the data show that the data collection, monitoring and reporting aspects if the trial delivery were feasible and acceptable; and (3) we were unable to we demonstrate that is it possible to recruit participants to this trial, suggesting that it is difficult to do within the NHS setting.

Recruitment to RCTs is challenging and it is important to report the root causes of recruitment difficulties so that mistakes are not repeated in future studies. Emerging data was explored by the trial team to consider how to problem solve and optimise procedures.

The number of patients fitted with a HA who were followed-up and screened for eligibility (n=466), was extremely low in comparison to the number of expected HA fittings (>4000). The study sites' data reporting mechanisms struggled to accurately report the patient flow (monthly HA fittings)

For peer review only - http://bmjopen.bmj.com/site/about/guidelines.xhtml

through this pathway but actual non-attendance rates for patients offered a follow-up appointment were calculated to be 30%, the level planned for, and do not account for this short fall.

During the study planning phase, both sites agreed to introduce face-to-face or telephone follow-up (not previously offered, despite NICE guidelines indicating this as good practice [43]) for all patients in the TAU pathway. The low number of patients seen for follow-up and then screened for eligibility by both sites suggests fully embedding and scaling-up this change took longer in practice than anticipated. Not all patients in the pathway were offered post-fitting follow-up and not all who attended were screened, leading to a significantly smaller pool of potential participants to screen (problems also highlighted by Donovan et al. [44]).

There is some evidence that RCTs with more restrictive eligibility criteria exhibit poorer recruitment than those with wider criteria.[45] After 6 weeks of missed monthly recruitment targets, we reviewed our eligibility criteria. As noted previously, the relationship between daily hours of HA use and HA outcomes is not well specified.[46] Consequently, criterion Q1 (hours of use), was dropped and the level of benefit (Q2) criterion widened to include patients reporting moderate benefit. Audiologists' feedback supported these changes and by the end of recruitment over 26% of those screened had identified as struggling. We had factored in a conservative estimate of 10%, based on clinical experience at each study site. The disparity reflects the modified eligibility criteria and variability in self-reported HA use data noted earlier.

Despite these problems, the screening process appeared to successfully identify the target population since the demographic profile of those screened and consented was as expected. The range of ages (64.1 – 82.1) indicated that it was older, not younger participants who were both

For peer review only - http://bmjopen.bmj.com/site/about/guidelines.xhtml

eligible and willing to participant. Their hearing profile was consistent with that expected from struggling hearing aid users.

Our a priori estimate (30%) of willing participants was closer to 14%. Since willingness is dependent on project specifics, population demographics, and patient perceptions of the research's value and worth, this was a feasibility parameter of interest. We could find no relevant study reporting detailed recruitment statistics in this context for comparison.

Comparing our recruitment issues with earlier ACE studies [27,29] is difficult since their context was very different, not least because they recruited from an already motivated population, rather than a specific publicly funded pathway known to foster motivational challenges, as reported here. Descriptions of the burden associated with RCT participation include, anxiety about taking part in research, factors related to processing trial information, demanding follow-up, and lack of appropriate post-trial management.[47] Our patients were possibly already feeling overwhelmed by managing their new HA and were less motivated to engage in research. Of the eligible, non-consenting patients, the majority gave no reason, were not interested or were too busy. Hickson et al [28] reported a much lower non-consent rate (20% compared to 86% here) and none of the burdens identified by Naidoo and colleagues [47] were overtly apparent.

It is notable that patients' decisions to not take part were made at clinical follow-up; few were passed on to the Research Nurse for further information and consent. Audiologists reported struggling to find time to answer participants' questions fully (either by phone or face-to-face). Thus, the opportunity for prospective participants to process the trial information at this point may not

For peer review only - http://bmjopen.bmj.com/site/about/guidelines.xhtml

have been optimum, and they declined referral to the Research Nurse. However, this was not reflected in the patients' narrative on non-consenting.

Research involvement can also be burdensome for clinicians and time is a frequently voiced barrier to their participation in research.[48,49] Pressures from usual clinical practice, [48,50] time demands of recruitment and follow-up, leading to increases in workload pressure [51] are associated with under-recruitment, as is a lack of research experience, [48,52] which was something the interviews with the screening audiologists revealed as a critical concern.

During the project development, audiologists and service managers said they could adapt their practice to include ACE screening. However, over time, they considered the screening questionnaire to be onerous and one site felt that they required more help from the "University Team" to answer patient's questions, despite having a dedicated research nurse/practitioner on site.

It is unlikely that patients declined to participate because they thought the research unimportant since participant interviews and pre-study PPI work indicated our research question was perceived as important.

Important aspects of recruitment such as ensuring simple procedures for information provision and consenting, and that study-related follow-ups do not increase pressure on usual clinical follow-ups (either for clinicians or patients) [52,53] were not really tested here because so few participants consented. Interviews with those who did indicated these aspects were not off-putting.

For peer review only - http://bmjopen.bmj.com/site/about/guidelines.xhtml

Many aspects of the study processes worked well. The randomisation process was viewed as acceptable by the small cohort recruited although insufficient numbers were achieved to allocate the last four participants recruited. There was no sense from the recruitment process that SO recruitment was problematic or that including SOs had a detrimental effect on recruitment. The SO/participant ratio (albeit from minimal data) is commensurate with similar studies [15,28] and was viewed as a positive incentive by participants. Having a SO was not an inclusion criterion, not disadvantaging those without a SO.

The trial processes undertaken for collecting outcome measures worked well. Outcome measure completion rates and interview data suggests that processes worked and are acceptable to participants. Due to insufficient participant numbers, estimation of SD for the PROMS, quality of life tools and healthcare resource use questionnaires was not possible. Completion and retention rates both indicate that whilst we were unable to recruit into the trial, those who were randomised stayed involved, suggesting a trial may have been possible had recruitment not been an issue. Attempts to increase recruitment via amendments to the study paperwork and processes failed, and further study site training proved unsuccessful. A plan to add more centres, change the point at which HA users were approached to the fitting appointment, and to transfer all responsibility for recruitment from the audiologists to the research team was ethically approval. However, although the funder granted the additional time required, extra funding was refused.

The ACE delivery processes appeared to work well, with the facilitator becoming more flexible over the five sessions to accommodate participant's needs within the ACE protocol. Participants mostly completed the programme and were generally positive in their feedback, citing changes they were making. This is in line with previous studies using ACE in Australia, Sweden and Chile.[28,30,54] All

For peer review only - http://bmjopen.bmj.com/site/about/guidelines.xhtml

previous studies reported good adherence to the ACE program (>90% completed 3 or more of the 5 sessions), and positive outcomes were indicated in terms of communication. All studies found pre-post ACE improvements in communication strategy use, particularly for those with mild hearing loss; activity and participation and psychosocial well-being, with findings reflected in positive feedback. Despite the small numbers of participants in this current study, participants also reported positive benefits from completing the ACE programme. A key challenge to the efficacy of delivering ACE here was the small group sizes that limited group discussion, so it was interesting and encouraging to note the broad changes people made in their communication strategies because the ACE programme.

CONCLUSION

A future RCT using the recruitment strategy reported here is not likely to be feasible. The fundamental problem was in accessing sufficient struggling hearing aid users at follow-up. Recruiting hearing aid users when first fitted may be a better strategy for future research. Additional work to investigate which point in the pathway patients should best be approached and to identify the resources needed for good recruitment is required. Work to develop a robust service delivery model of follow-up for new adult HA users based on NICE guidelines will be crucial, as would implementing organisational support for NHS audiologists involved in research.

Acknowledgments

This work uses data provided by patients and collected by the NHS as part of their care and support and would not have been possible without access to this data. The Authors would also like to thank the members of the Project Advisory Panel (PAP), Emmanuelle Blondiaux-Ding, Philip Le Mare and Shona Hudson for their invaluable advice on many aspects of the project.

For peer review only - http://bmjopen.bmj.com/site/about/guidelines.xhtml

Contributors

NJT led on the original conception and design of the study with substantial contributions to the protocol from JW, CJ, EC, AF, LH, KB, CF, CH, CM, LG, RG and KI. As Chief Investigator, NJT takes overall responsibility for the work. CM liaised with the participants, delivered the ACE intervention and oversaw the PAP meetings. JW made substantial contributions to the trial design and management and manuscript drafting. EC was responsible for data analysis and data interpretation. KB was responsible for the health economic design and analysis. CF and CH provided further statistical advice and expertise on the study. CJ was responsible for qualitative interviews and data collection and transcript analysis. AF was involved in all aspects of the study and KI and RG were recruiting site leads, responsible for aspects specific to Trust's service delivery and providing expert clinical support. All authors contributed to draft manuscripts, read and approved the final manuscript.

Approvals obtained

Ethical approval was granted by South East Coast-Surrey Research Ethics Committee (16/LO/2012) on 02nd December 2016 and Health Research Authority approval was obtained on 16th January 2017.

Funding Statement

This paper presents independent research funded by the National Institute for Health Research (NIHR) under its Research for Patient Benefit (RfPB) Programme (Grant Reference Number PB-PG-0215-36147). The views expressed are those of the author(s) and not necessarily those of the NHS, the NIHR or the Department of Health and Social Care.

Patient consent for publication

For peer review only - http://bmjopen.bmj.com/site/about/guidelines.xhtml

Not required

Competing Interests

None

Data Availability Statement

The data that support the findings of this study are available from the corresponding author, [JW], upon reasonable request.

REFERENCES

1. Organisation WH. WHO global estimates on prevalence of hearing loss: Mortality and Burden of Diseases and Prevention of Blindness and Deafness. Secondary WHO global estimates on prevalence of hearing loss: Mortality and Burden of Diseases and Prevention of Blindness and Deafness. 2012. http://www.who.int/pbd/deafness/WHO_GE_HL.pdf.
2. Davis AC. The Prevalence of Hearing Impairment and Reported Hearing Disability Among Adults in Great Britain. *Int J of Epidemiol* 1989;18(4):911-17.
3. AoHL. Hearing Matters: Taking action on hearing loss in the 21st century. http://www.actiononhearingloss.org.uk/supporting-you/policy-research-and-influencing/research/hearing-matters.aspx: Action on Hearing Loss, 2011.
4. Roth TN, Hanebuth D, Probst R. Prevalence of age-related hearing loss in Europe: a review. *Eur Arch Otorhinolaryngol* 2011;268(8):1101-07.
5. Bess FH, Lichtenstein MJ, Logan SA, et al. Hearing impairment as a determinant of function in the elderly. *J Am Geriatr Soc* 1989;37(11):123-28.
6. Chia EM, Wang JJ, Rochtchina E, et al. Hearing impairment and health-related quality of life: The blue mountains hearing study. *Ear Hear* 2007;28:187-95.
7. Health Quality Ontario. Social isolation in community-dwelling seniors: an evidence-based analysis. *Ont Health Technol Assess Ser* 2008;8(5):1-49.
8. Pronk M, Deeg DJH, Smits C, et al. Prospective effects of hearing status on loneliness and depression in older persons: Identification of subgroups. *Int J Audiol* 2011, 50(12): 887-896.
9. Hetu R, Jones L, Getty L. The impact of acquired hearing impairment on intimate-relationships - implications for rehabilitation. *Audiology* 1993;32(6):363-81.
10. Heine C, Browning C. The communication and psychosocial perceptions of older adults with sensory loss: a qualitative study. *Ageing Soc* 2004;24:113-30.

For peer review only - http://bmjopen.bmj.com/site/about/guidelines.xhtml

11. Heine C, Browning CJ. Communication and psychosocial consequences of sensory loss in older adults: overview and rehabilitation directions. *Disabil Rehabil* 2002;24(15):763-73.

12. Humes LE, Dubno JR, Gordon-Salant S, et al. Central Presbycusis: A Review and Evaluation of the Evidence. *J Am Acad Audiol* 2012;23(8):635-66.

13. Stephens D, Kramer SE. Living with hearing loss: The Process of Enablement. London: Wiley, 2009.

14. Scarinci N, Worrall L, Hickson L. The effect of hearing impairment in older people on the spouse: Development and psychometric testing of The Significant Other Scale for Hearing Disability (SOS-HEAR). *Int J Audiol* 2009;48(10):671-83.

15. Scarinci N, Worrall L, Hickson L. Factors Associated With Third-Party Disability in Spouses of Older People With Hearing Impairment. *Ear Hear* 2012;33(6):698-708.

16. Wallhagen MI, Strawbridge WJ, Shema SJ, et al. Impact of self-assessed hearing loss on a spouse: A longitudinal analysis of couples. *J Gerontol B Psychol Sci Soc Sci* 2004;59(3):S190-S96.

17. Brooks DN, Hallam RS, Mellor PA. The effects on significant others of providing a hearing aid to the hearing-impaired partner. *Br J Audiol* 2001; 35: 165–171

18. Kochkin S. MarkeTrak VIII patients report improved quality of life with hearing aid usage. *Hearing J* 2011;64(6):25-32.

19. Laplante-Levesque A, Hickson L, Worrall L. Rehabilitation of Older Adults With Hearing Impairment: A Critical Review. *J Aging Health* 2010;22(2):143-53.

20. Ferguson MA, Kitterick PT, Chong LY, et al. Hearing aids for mild to moderate hearing loss in adults. *Cochrane Database Syst Rev* 2017(9).

21. Gianopoulos I, Stephens D, Davis A. Follow up of people fitted with hearing aids after adult hearing screening: the need for support after fitting. *BMJ* 2002;325.

22. Hougaard S, Ruf S. EuroTrak I: A consumer survey about hearing aids in Germany, France and the UK. First global comparative study of hearing, hearing loss, and hearing aids. The *Hearing Rev* 2011;18(2):12-28.

23. Stephens D, Lewis P, Davis A, et al. Hearing aid possession in the population: Lessons from a small country. *Audiology* 2001;40(2):104-11.

24. Ng JH-Y, Loke AY. Determinants of hearing aid adoption and use among the elderly: A systematic review. *Int J Audiol* 2015;54(5):291-300.

25. Gagne JP, Jennings MB, Southall K. The ICF: A Classification System and Conceptual Framework Ideal for Audiological Rehabilitation. *Perspectives on Aural Rehabilitation and its Instrumentation* 2009;16(1):8-14.

26. Lockey K, Jennings MB, Shaw L. Exploring hearing aid use in older women through narratives. *Int J Audiol* 2010;49(8):542-49.

27. Hickson L, Worrall L, Scarinci N, editors. *ACE: Active Communication Education.* University of Queensland, 2015.

For peer review only - http://bmjopen.bmj.com/site/about/guidelines.xhtml

28. Hickson L, Worrall L, Scarinci N. A randomized control trial evaluating the Active Communication Education program for older people with hearing impairment. *Ear Hear* 2007;28(2):212-30.

29. Oberg M, Bohn T, Larsson U. Short- and Long-Term Effects of the Modified Swedish Version of the Active Communication Education (ACE) Program for Adults with Hearing Loss. *J Am Acad Audiol* 2014;25(9):848-58.

30. Rivera S, Marcotti A, Fuente A, et al. Adaptation of the "Active Communication Education" program into Spanish for older adults with hearing loss. *Int J Audiol* 2020. doi:10.1080/14992027.2020.1740801

31. Thyer NJ, Watson J, Jackson C, et al. Randomised controlled feasibility trial of the Active Communication Education programme plus hearing aid provision versus hearing aid provision alone (ACE to HEAR): a study protocol. *BMJ Open* 2018;8:e021502. doi:10.1136/ bmjopen-2018-021502

32. Cox RM, Alexander GC. The International Outcome Inventory for Hearing Aids (IOI-HA): psychometric properties of the English version. *Int J Audiol* 2002;41(1):30-35.

33. Schow RL, Nerbonne MA. Communication screening profile - use with elderly clients. *Ear Hear* 1982;3(3):135-47.

34. Herdman M, Gudex C, Lloyd A, et al. Development and preliminary testing of the new five-level version of EQ-5D (EQ-5D-5L). Quality of Life Research. *Qual Life Res* 2011;20(10):1727-36.

35. Janssen M, Pickard A, Golicki D, et al. Measurement properties of the EQ-5D-5L compared to the EQ-5D-3L across eight patient groups: a multi-country study. *Qual Life Res* 2013;22(7):1717-27.

36. Ware J, Sherbourne C. The MOS 36-item short-form health survey (SF-36) 1: conceptual framework and item selection. *Med Care* 1992;30:473-83.

37. Bellg AJ, Resnick B, Minicucci DS, et al. Enhancing Treatment Fidelity in Health Behavior Change Studies: Best Practices and Recommendations From the NIH Behavior Change Consortium. *Health Psychol* 2004;23(5):441-51.

38. Noble W. Extending the IOI to significant others and to non-hearing aid-based interventions. *Int J Audiol* 2002;41(1):27-29.

39. National Institute of Health and Clinical Excellence [NICE]. Guide to the methods of technology appraisal (PMG9). London: NICE 2013.

40. Ritchie J, Lewis J. *Qualitative Research Practice*. London: Sage Publications, 2003.

41. Curtis L, Burns A (2018) Unit Costs of Health and Social Care 2018, Personal Social Services Research Unit, University of Kent, Canterbury. https://doi.org/10.22024/UniKent/01.02.70995

42. Department of Health. NHS Reference Costs 2017/18. London: NICE 2018.

43. National Institute of Health and Clinical Excellence [NICE]. Hearing loss in adults: assessment and management (NG98). London, 2018.

For peer review only - http://bmjopen.bmj.com/site/about/guidelines.xhtml

44. Donovan JL, Paramasivan S, de Salis I, *et al.* Clear obstacles and hidden challenges: understanding recruiter perspectives in six pragmatic randomised controlled trials. *Trials* **15,** 5 (2014). https://doi.org/10.1186/1745-6215-15-5

45. Briel M, Speich B, von Elm E et al. Comparison of randomized controlled trials discontinued or revised for poor recruitment and completed trials with the same research question: a matched qualitative study. *Trials* 2019; **20:**800.

46. Perez E, Edmonds BA. A Systematic Review of Studies Measuring and Reporting Hearing Aid Usage in Older Adults since 1999: A Descriptive Summary of Measurement Tools. *PLoS ONE* 2012; 7(3): e31831. https://doi.org/10.1371/journal.pone.0031831

47. Naidoo N, Nguyen VT, Ravaud P et al. The research burden of randomized controlled trial participation: a systematic thematic synthesis of qualitative evidence. *BMC Med* 2020; 18;6 https://doi.org/10.1186/s12916-019-1476-5

48. Bullen T, Maher K, Rosenberg JP et al. Establishing research in a palliative care clinical setting: Perceived barriers and implemented strategies. *App Nurs Res* 2014*; 27*: 78-83 https://doi.org/10.1016/j.apnr.2013.10.007

49. Harding KE, Porter J, Horne-Thompson A et al. Not enough time or a low priority? Barriers to evidence-based practice for allied health clinicians. *J Contin Educ Health Prof* 2014*;34*(4): 224-231. https://doi.org/10.1002/chp

50. Messner DA, Moloney R, Warriner AH, et al. Understanding practice-based research participation: The differing motivations of engaged vs. non-engaged clinicians in pragmatic clinical trials. *Contemp Clin Trials Commun* 2016; 4, 136-140. https://doi.org/10.1016/j.conctc.2016.08.003

51. Paget SP, Caldwell PHY, Murphy J, et al. (2017). Moving beyond 'not enough time': factors influencing paediatric clinicians' participation in research. *Intern Med J, 47*(3), 299-306. https://doi.org/10.1111/imj.13351

52. Isaksson E, Wester P, Laska AC *et al.* Identifying important barriers to recruitment of patients in randomised clinical studies using a questionnaire for study personnel. *Trials* **20,** 618 (2019). https://doi.org/10.1186/s13063-019-3737-1

53. Caldwell PH, Hamilton S, Tan A et al. Strategies for increasing recruitment to randomised controlled trials: systematic review. *PLoS Medicine 2010; 7*(11):e1000368. https://doi.org/10.1371/journal.pmed.1000368

54. Öberg M. A multicentre study evaluating the effects of the Swedish ACE programme. *Int J Audiol* 2017;56: 876-886.

For peer review only - http://bmjopen.bmj.com/site/about/guidelines.xhtml

Figure 1: Consort flow diagram

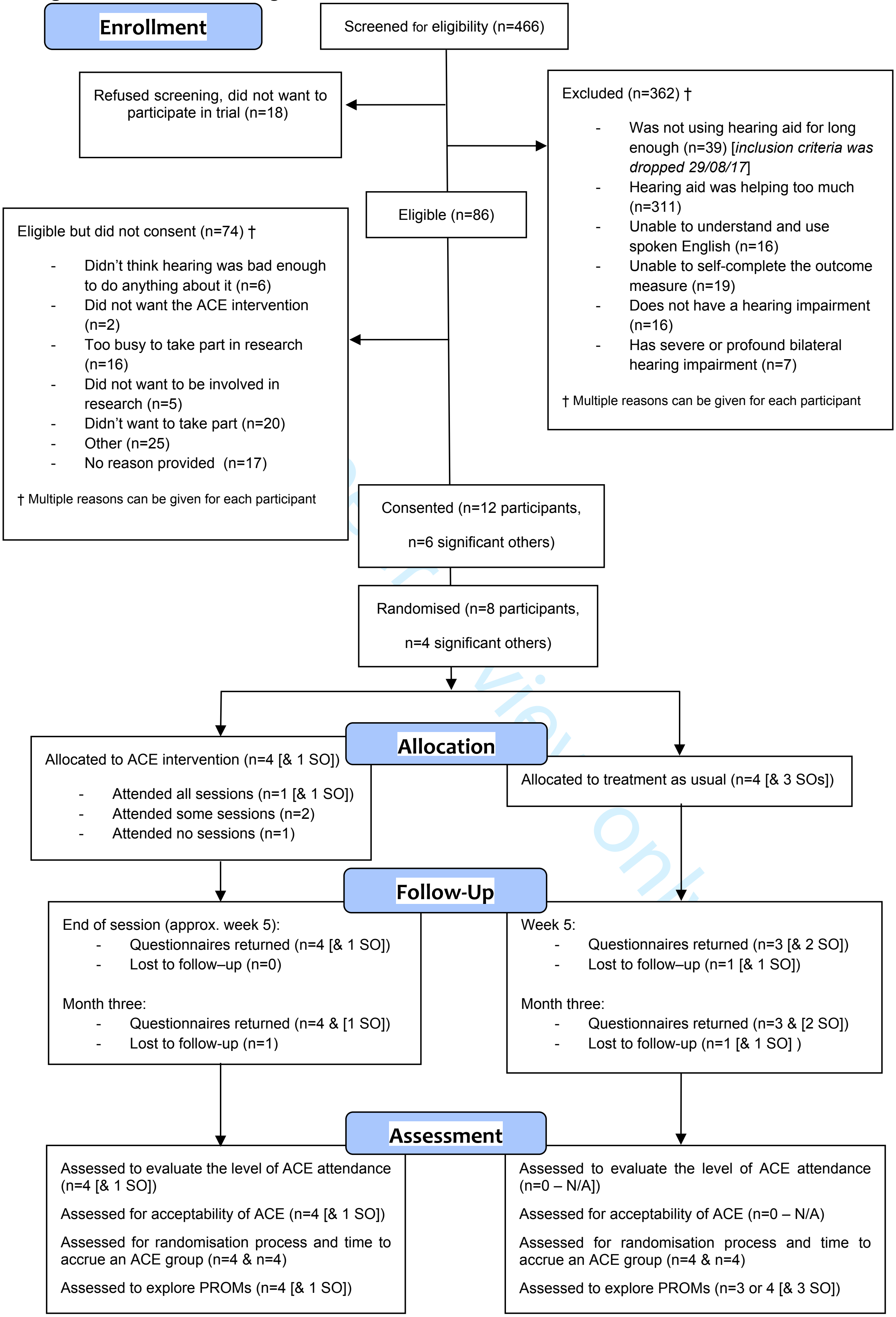

For peer review only - http://bmjopen.bmj.com/site/about/guidelines.xhtml

Supplementary material for manuscript:

A randomised controlled feasibility trial of an Active Communication Education programme plus hearing aid provision versus hearing aid provision alone (ACE To HEAR)

Figure A: Monthly recruitment to the study, by site

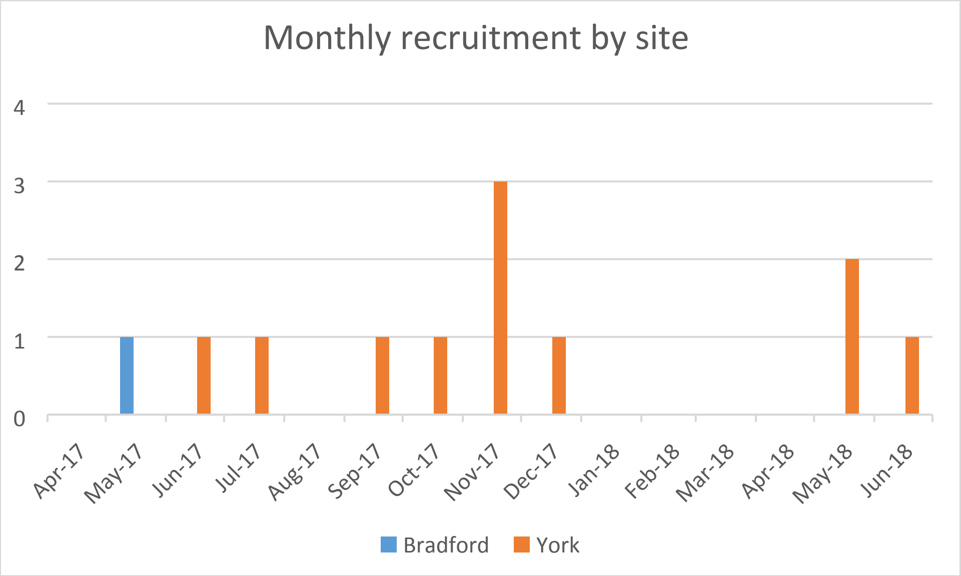

Table A: Hearing aid use, hearing thresholds and aetiology for the randomised participants by group and overall

	**ACE Intervention (n=4)**	**Control (n=4)**	**All (n=8)**
**Average daily hearing aid use over the past two weeks:**			
None	1 (25.0)	0 (0.0)	1 (12.5)
Less than 1 hour a day	0 (0.0)	0 (0.0)	0 (0.0)
Between 1 and 4 hours a day	1 (25.0)	3 (75.0)	4 (50.5)
Between 4 and 8 hours a day	0 (0.0)	0 (0.0)	0 (0.0)
More than 8 hours a day	2 (50.0)	1 (25.0)	3 (37.5)

For peer review only

**Level of help from hearing aid in the last two weeks:**			
Helped not at all	1 (25.0)	0 (0.0)	1 (12.5)
Helped slightly	1 (25.0)	1 (25.0)	2 (25.0)
Helped moderately	2 (50.0)	3 (75.0)	5 (62.5)
Helped quite a lot	0 (0.0)	0 (0.0)	0 (0.0)
Helped very much	0 (0.0)	0 (0.0)	0 (0.0)
Hearing Thresholds			
	N=4	N=4	N=8
Left Ear	27.5 (9.6)	18.8 (8.5)	23.1 (9.6)
**500Hz**	30 (15, 35)	17.5 (10, 30)	22.5 (10, 35)
Mean (SD)			
Median (min, max)			
	40 (20.4)	26.3 (13.8)	33.1 (17.7)
**1000Hz**	40 (15, 65)	27.5 (10, 40)	37.5 (10, 65)
Mean (SD)			
Median (min, max)			
	61.3 (11.1)	41.3 (8.5)	51.3 (14.1)
**2000Hz**	60 (50, 75)	42.5 (30, 50)	50 (30, 75)
Mean (SD)			
Median (min, max)			
	75 (9.1)	52.5 (10.4)	63.8 (15.1)
**4000Hz**	75 (65, 85)	52.5 (40, 65)	65 (40, 85)
Mean (SD)			
Median (min, max)			
	73.8 (23.2)	70 (9.2)	71.9 (16.5)
**8000Hz**	77.5 (45, 95)	70 (60, 80)	70 (45, 95)
Mean (SD)			
Median (min, max)			
Right Ear			
**500Hz**	N=4	N=4	N=8
Mean (SD)	26.3 (18.9)	22.5 (8.7)	24.4 (13.7)
Median (min, max)	25 (10, 45)	20 (15, 35)	20 (10, 45)
**1000Hz**			
Mean (SD)	36.3 (15.5)	32.5 (15.5)	34.4 (14.5)
Median (min, max)	40 (15, 50)	37.5 (10, 45)	37.5 (10, 50)
**2000Hz**			
Mean (SD)	41.3 (7.5)	52.5 (22.2)	46.9 (16.5)
Median (min, max)	45 (30, 45)	50 (30, 80)	45 (30, 80)
**4000Hz**			

For peer review only

Mean (SD)<br>Median (min, max)	75 (14.8)<br>75 (60, 90)	63.8 (19.3)<br>62.5 (45, 85)	69.4 (17.0)<br>70 (45, 90)
**8000Hz**<br>Mean (SD)<br>Median (min, max)	80 (23.8)<br>90 (45, 95)	71.3 (25.0)<br>65 (50, 105)	75.6 (23.1)<br>80 (45, 105)
**Aetiology, n (%)**			
Sensorineural	3 (75.0)	4 (100.0)	7 (87.5)
Conductive	0 (0.0)	0 (0.0)	0 (0.0)
Mixed	1 (25.0)	0 (0.0)	1 (12.5)
Other	0 (0.0)	0 (0.0)	0 (0.0)

Table B: International Outcomes Inventory for Hearing Aids (IOI-HA) results for participants, by group and overall (Higher score is better, range 7-35)

	ACE Intervention participants (n=4)	Control participants (n=4)	All participants (n=8)
**Baseline**<br>Mean (SD)<br>Median (min, max)	N=4<br>22.5 (6.6)<br>23 (15, 29)	N=3<br>25.7 (3.1)<br>25 (23, 29)	N=7<br>23.9 (5.3)<br>25 (15, 29)
**Week 5**<br>Mean (SD)<br>Median (min, max)	N=3<br>23.0 (3.6)<br>24 (19, 26)	N=3<br>26.3 (4.7)<br>28 (21, 30)	N=6<br>24.7 (4.2)<br>25 (19, 30)
**3 months post-randomisation**<br>Mean (SD)<br>Median (min, max)	N=3<br>20 (1.7)<br>19 (19, 22)	N=3<br>27.7 (3.5)<br>28 (24, 31)	N=6<br>23.9 (4.9)<br>23 (19, 31)

Table C: Self-Assessment of Communication (SAC) (Lower score is better, range 0 – 100)

	ACE Intervention participants (n=4)	Control Participants (n=4)	All participants (n=8)
**Baseline**<br>Mean (SD)<br>Median (min, max)	N=4<br>65 (21.9)<br>58.8 (47.5, 95)	N=4<br>20 (7.9)<br>21.3 (10, 27.5)	N=8<br>42.5 (28.5)<br>37.5 (10, 95)
**3 months post-randomisation**<br>Mean (SD)<br>Median (min, max)	N=3<br>62.5 (10.9)<br>57.5 (55, 75)	N=3<br>22.5 (4.3)<br>25 (17.5, 25)	N=6<br>42.5 (23.1)<br>40 (17.5, 75)

For peer review only

Table D: International Outcomes Inventory for Alternative Interventions (IOI-AI) (scored 1 – 5, higher score is better)

	ACE intervention participants (n=4)
**Week 5**	N=3
Mean (SD)	2.6 (0.7)
Median (min, max)	3 (1.9, 3.1)
**3 months post-randomisation**	N=2
Mean (SD)	2.6 (0.6)
Median (min, max)	2.6 (2.1, 3)

Table E: International Outcomes Inventory for Hearing Aids: version for Significant Others (IOI-HA-SO) results for significant others, overall (Higher score is better, range 7 - 35)

	All SOs (n=4)
**Baseline**	N=4
Mean (SD)	23.8 (5.3)
Median (min, max)	24 (17, 30)
**Week 5**	N=3
Mean (SD)	25.7 (3.1)
Median (min, max)	25 (23, 29)
**3 months post-randomisation**	N=3
Mean (SD)	24 (4.0)
Median (min, max)	24 (20, 28)

Table F: Significant Other Scale for Hearing Disability (SOS-HEAR) (scored 0 – 4, higher scores indicate greater difficulties experienced by the SO.)

	All SOs (n=4)
**Baseline**	N=4
Mean (SD)	1.0 (0.5)
Median (min, max)	0.9 (0.5, 1.8)
**Week 5**	N=3
Mean (SD)	1.2 (1.0)
Median (min, max)	0.8 (0.5, 2.3)
**3 months post-randomisation**	N=3
Mean (SD)	1.4 (1.0)
Median (min, max)	1.1 (0.6, 2.4)

For peer review only

Table G: Average health care resource use by trial arm at baseline

	ACE				TAU			
	n	Mean (SD)	Min, Max	Missing	n	Mean (SD)	Min, Max	Missing
**GP Visit**	2	0.5 0.71	0 1	2	4	0	0, 0	0
**home**	2	0 (0)	0, 0	2	4	0 (0)	0, 0	0
**Physiotherapist**	2	0 (0)	0, 0	2	4	0 (0)	0, 0	0
**Nurse**	2	0 (0)	0, 0	2	4	0 (0)	0, 0	0
**District/ community nurse**	0	0 (0)	0, 0		4	0 (0)	0, 0	0
**Hearing therapist**	2	1 (1.41)	0, 2	2	3	0.33 (0.58)	0, 1	1
**Speech therapist**	2	0 (0)	0, 0	2	4	0 (0)	0, 0	0
**Counsellor**	2	0 (0)	0, 0	2	4	0 (0)	0, 0	0
**Psychologist**	2	0 (0)	0, 0	2	4	0 (0)	0, 0	0
**Psychiatrist**	2	0 (0)	0, 0	2	4	0 (0)	0, 0	0
**ENT nurse**	2	0 (0)	0, 0	2	4	0.75 (1.5)	0, 3	0
**ENT doctor**	2	0 (0)	0, 0	2	4	0.75 (1.5)	0, 3	0
**Outpatient attendance**	2	1.5 (0.71)	1 2	2	4	1.75 (1.71)	0, 4	0
**A&E attendance**	2	0 (0)	0, 0	2	4	0 (0)	0, 0	0
**Day case**	2	0.5 (0.71)	0, 1	2	4	0 (0)	0, 0	0
**In patient per night**	2	0 (0)	0, 0	2	4	0 (0)	0, 0	0
**Private physiotherapist**	2	0 (0)	0, 0	2	4	0 (0)	0, 0	0
**Private audiologist**	2	0 (0)	0, 0	2	4	0 (0)	0, 0	0
**Private osteopath**	2	0 (0)	0, 0	2	4	0 (0)	0, 0	0
**Private counsellor**	2	0 (0)	0, 0	2	4	0 (0)	0, 0	0
**Private acupuncturist**	2	0 (0)	0, 0	2	4	0 (0)	0, 0	0
**Private chiropractitioner**	2	0 (0)	0, 0	2	4	0 (0)	0, 0	0
**Private day case**	2	0 (0)	0, 0	2	4	0 (0)	0, 0	0
**Private in patient per night**	2	0 (0)	0, 0	2	4	0 (0)	0, 0	0

For peer review only

Table H: Average health care resource use by trial arm at 3 months post-randomisation

	ACE				TAU			
	**n**	**Mean (SD)**	**Min, Max**	**Missing**	**n**	**Mean (SD)**	**Min, Max**	**Missing**
**GP Visit**	1	0 (0)	0, 0	3	3	0 (0)	0, 0	1
**home**	1	0 (0)	0, 0	3	3	0 (0)	0, 0	1
**Physiotherapist**	1	0 (0)	0, 0	3	3	0 (0)	0, 0	1
**Nurse**	0	-	-	4	3	0 (0)	0, 0	1
**District/ community nurse**	1	0 (0)	0, 0	3	3	0 (0)	0, 0	1
**Hearing therapist**	2	0.5 (0.71)	0, 1	2	3	0 (0)	0, 0	1
**Speech therapist**	1	0 (0)	0, 0	3	3	0 (0)	0, 0	1
**Counsellor**	1	0 (0)	0, 0	3	3	0 (0)	0, 0	1
**Psychologist**	1	0 (0)	0, 0	3	3	0 (0)	0, 0	1
**Psychiatrist**	1	0 (0)	0, 0	3	3	0 (0)	0, 0	1
**ENT nurse**	1	1 (-)	1, 1	3	3	0 (0)	0, 0	1
**ENT doctor**	1	0 (0)	0, 0	3	3	0 (0)	0, 0	1
**Outpatient attendance**	1	1 (-)	1, 1	3	3	0 (0)	0, 0	1
**A&E attendance**	1	0 (0)	0, 0	3	3	0 (0)	0, 0	1
**Day case**	1	0 (0)	0, 0	3	3	0 (0)	0, 0	1
**In patient per night**	1	0 (0)	0, 0	3	3	0 (0)	0, 0	1
**Private physiotherapist**	1	0 (0)	0, 0	3	3	0 (0)	0, 0	1
**Private audiologist**	1	2 (-)	2, 2	3	3	0 (0)	0, 0	1
**Private osteopath**	1	0 (0)	0, 0	3	3	0 (0)	0, 0	1
**Private counsellor**	1	0 (0)	0, 0	3	3	0 (0)	0, 0	1
**Private acupuncturist**	1	0 (0)	0, 0	3	3	0 (0)	0, 0	1
**Private chiropractitioner**	1	0 (0)	0, 0	3	3	0 (0)	0, 0	1
**Private day case**	1	0 (0)	0, 0	3	3	0 (0)	0, 0	1
**Private in patient per night**	1	0 (0)	0, 0	3	3	0 (0)	0, 0	1

For peer review only

CONSORT 2010 checklist of information to include when reporting a pilot or feasibility randomized trial in a journal or conference abstract

Item	Description	Reported on manuscript line number
Title	Identification of study as randomised pilot or feasibility trial	1
Authors *	Contact details for the corresponding author	28
Trial design	Description of pilot trial design (eg, parallel, cluster)	36-37
Methods		
Participants	Eligibility criteria for participants and the settings where the pilot trial was conducted	38; 39-40
Interventions	Interventions intended for each group	41-43
Objective	Specific objectives of the pilot trial	33-35
Outcome	Prespecified assessment or measurement to address the pilot trial objectives**	44-46
Randomization	How participants were allocated to interventions	42
Blinding (masking)	Whether or not participants, care givers, and those assessing the outcomes were blinded to group assignment	48-49
Results		
Numbers randomized	Number of participants screened and randomised to each group for the pilot trial objectives**	50-51; 53
Recruitment	Trial status†	NA
Numbers analysed	Number of participants analysed in each group for the pilot objectives**	50-51
Outcome	Results for the pilot objectives, including any expressions of uncertainty**	55-56
Harms	Important adverse events or side effects	Main text 353
Conclusions	General interpretation of the results of pilot trial and their implications for the future definitive trial	57-59
Trial registration	Registration number for pilot trial and name of trial register	60
Funding	Source of funding for pilot trial	Main text 560

Citation: Eldridge SM, Chan CL, Campbell MJ, Bond CM, Hopewell S, Thabane L, et al. CONSORT 2010 statement: extension to randomised pilot and feasibility trials. BMJ. 2016;355.

**this item is specific to conference abstracts*

***Space permitting, list all pilot trial objectives and give the results for each. Otherwise, report those that are a priori agreed as the most important to the decision to proceed with the future definitive RCT.*

†For conference abstracts.

For peer review only

CONSORT 2010 checklist of information to include when reporting a pilot or feasibility trial*

Section/Topic	Item No	Checklist item	Reported on page No
**Title and abstract**			
	1a	Identification as a pilot or feasibility randomised trial in the title	1
	1b	Structured summary of pilot trial design, methods, results, and conclusions (for specific guidance see CONSORT abstract extension for pilot trials)	3
**Introduction**			
Background and objectives	2a	Scientific background and explanation of rationale for future definitive trial, and reasons for randomised pilot trial	5-6
	2b	Specific objectives or research questions for pilot trial	6-7
**Methods**			
Trial design	3a	Description of pilot trial design (such as parallel, factorial) including allocation ratio	6
	3b	Important changes to methods after pilot trial commencement (such as eligibility criteria), with reasons	7-8
Participants	4a	Eligibility criteria for participants	3; 7
	4b	Settings and locations where the data were collected	6
	4c	How participants were identified and consented	6
Interventions	5	The interventions for each group with sufficient details to allow replication, including how and when they were actually administered	6
Outcomes	6a	Completely defined prespecified assessments or measurements to address each pilot trial objective specified in 2b, including how and when they were assessed	8-10
	6b	Any changes to pilot trial assessments or measurements after the pilot trial commenced, with reasons	-
	6c	If applicable, prespecified criteria used to judge whether, or how, to proceed with future definitive trial	9-10
Sample size	7a	Rationale for numbers in the pilot trial	7
	7b	When applicable, explanation of any interim analyses and stopping guidelines	-
Randomisation:			
Sequence generation	8a	Method used to generate the random allocation sequence	8
	8b	Type of randomisation(s); details of any restriction (such as blocking and block size)	8
Allocation concealment mechanism	9	Mechanism used to implement the random allocation sequence (such as sequentially numbered containers), describing any steps taken to conceal the sequence until interventions were assigned	8

For peer review only

Implementation	10	Who generated the random allocation sequence, who enrolled participants, and who assigned participants to interventions	8
Blinding	11a	If done, who was blinded after assignment to interventions (for example, participants, care providers, those assessing outcomes) and how	3
	11b	If relevant, description of the similarity of interventions	-
Statistical methods	12	Methods used to address each pilot trial objective whether qualitative or quantitative	10-11
**Results**			
Participant flow (a diagram is strongly recommended)	13a	For each group, the numbers of participants who were approached and/or assessed for eligibility, randomly assigned, received intended treatment, and were assessed for each objective	Fig 1; p12
	13b	For each group, losses and exclusions after randomisation, together with reasons	12-13
Recruitment	14a	Dates defining the periods of recruitment and follow-up	12
	14b	Why the pilot trial ended or was stopped	-
Baseline data	15	A table showing baseline demographic and clinical characteristics for each group	14
Numbers analysed	16	For each objective, number of participants (denominator) included in each analysis. If relevant, these numbers should be by randomised group	13-19
Outcomes and estimation	17	For each objective, results including expressions of uncertainty (such as 95% confidence interval) for any estimates. If relevant, these results should be by randomised group	13-22
Ancillary analyses	18	Results of any other analyses performed that could be used to inform the future definitive trial	13-22
Harms	19	All important harms or unintended effects in each group (for specific guidance see CONSORT for harms)	19
	19a	If relevant, other important unintended consequences	
**Discussion**			
Limitations	20	Pilot trial limitations, addressing sources of potential bias and remaining uncertainty about feasibility	22-27
Generalisability	21	Generalisability (applicability) of pilot trial methods and findings to future definitive trial and other studies	22-27
Interpretation	22	Interpretation consistent with pilot trial objectives and findings, balancing potential benefits and harms, and considering other relevant evidence	22-27
	22a	Implications for progression from pilot to future definitive trial, including any proposed amendments	27
**Other information**			
Registration	23	Registration number for pilot trial and name of trial registry	4
Protocol	24	Where the pilot trial protocol can be accessed, if available	6
Funding	25	Sources of funding and other support (such as supply of drugs), role of funders	28
	26	Ethical approval or approval by research review committee, confirmed with reference number	28

For peer review only

Citation: Eldridge SM, Chan CL, Campbell MJ, Bond CM, Hopewell S, Thabane L, et al. CONSORT 2010 statement: extension to randomised pilot and feasibility trials. BMJ. 2016;355.
*We strongly recommend reading this statement in conjunction with the CONSORT 2010, extension to randomised pilot and feasibility trials, Explanation and Elaboration for important clarifications on all the items. If relevant, we also recommend reading CONSORT extensions for cluster randomised trials, non-inferiority and equivalence trials, non-pharmacological treatments, herbal interventions, and pragmatic trials. Additional extensions are forthcoming: for those and for up to date references relevant to this checklist, see www.consort-statement.org.

For peer review only

BMJ Open

A randomised controlled feasibility trial of an Active Communication Education programme plus hearing aid provision versus hearing aid provision alone (ACE To HEAR)

Journal:	*BMJ Open*
Manuscript ID	bmjopen-2020-043364.R1
Article Type:	Original research
Date Submitted by the Author:	13-Dec-2020
Complete List of Authors:	Watson, Judith; University of York, Health Sciences; University of York<br>Coleman, Elizabeth; York Trials Unit, Health Sciences<br>Jackson, Cath; University of York, York Trials Unit<br>Bell, Kerry; University of York, Health Sciences<br>Maynard, Christina; Univerity of Leeds, Leeds Institute of Cardiovascular & Metabolic Medicine<br>Hickson, Louise; The University of Queensland, School of Health and Rehabilitation Sciences<br>Forster, Anne; University of Leeds, Academic Unit of Elderly Care and Rehabilitat<br>Fairhurst, Caroline; University of York, Health Sciences<br>Hewitt, Catherine; The University of York, Health Sciences<br>Gardner, Rob; Bradford Royal Infirmary, Audiology Department<br>Iley, Kate; York Teaching Hospital NHS Foundation Trust, Audiology<br>Gailey, Lorraine; Hearing Link<br>Thyer, Nicholas; Univerity of Leeds, Leeds Institute of Cardiovascular & Metabolic Medicine
**Primary Subject Heading**:	Ear, nose and throat/otolaryngology
Secondary Subject Heading:	Health services research, Research methods, Qualitative research
Keywords:	STATISTICS & RESEARCH METHODS, QUALITATIVE RESEARCH, Adult otolaryngology < OTOLARYNGOLOGY

SCHOLARONE™
Manuscripts

I, the Submitting Author has the right to grant and does grant on behalf of all authors of the Work (as defined in the below author licence), an exclusive licence and/or a non-exclusive licence for contributions from authors who are: i) UK Crown employees; ii) where BMJ has agreed a CC-BY licence shall apply, and/or iii) in accordance with the terms applicable for US Federal Government officers or employees acting as part of their official duties; on a worldwide, perpetual, irrevocable, royalty-free basis to BMJ Publishing Group Ltd ("BMJ") its licensees and where the relevant Journal is co-owned by BMJ to the co-owners of the Journal, to publish the Work in this journal and any other BMJ products and to exploit all rights, as set out in our licence.

The Submitting Author accepts and understands that any supply made under these terms is made by BMJ to the Submitting Author unless you are acting as an employee on behalf of your employer or a postgraduate student of an affiliated institution which is paying any applicable article publishing charge ("APC") for Open Access articles. Where the Submitting Author wishes to make the Work available on an Open Access basis (and intends to pay the relevant APC), the terms of reuse of such Open Access shall be governed by a Creative Commons licence – details of these licences and which Creative Commons licence will apply to this Work are set out in our licence referred to above.

Other than as permitted in any relevant BMJ Author's Self Archiving Policies, I confirm this Work has not been accepted for publication elsewhere, is not being considered for publication elsewhere and does not duplicate material already published. I confirm all authors consent to publication of this Work and authorise the granting of this licence.

For peer review only

A randomised controlled feasibility trial of an Active Communication Education programme plus hearing aid provision versus hearing aid provision alone (ACE To HEAR)

Judith Watson- York Trials Unit, Department of Health Sciences, University of York, ARRC Building, York, UK (Orcid iD 0000-0003-0694-3854)

Elizabeth Coleman - York Trials Unit, Department of Health Sciences, University of York, ARRC Building, York, UK (Orcid iD – 0000-0003-4210-1865)

Cath Jackson - Valid Research Ltd, Wetherby, UK (Orcid iD 0000-0003-3181-7091, https://uk.linkedin.com/in/cath-jackson-valid-research)

Kerry Bell- York Trials Unit, Department of Health Sciences, University of York, ARRC Building, York, UK (Orcid iD 0000-0001-5124-138X)

Christina Maynard - Leeds Institute of Cardiovascular and Metabolic Medicine (LICAMM,) University of Leeds, Leeds, UK and Bramhope Medical Centre, Leeds, UK

Louise Hickson - School of Health and Rehabilitation Sciences, University of Queensland, Brisbane, Queensland 4072, Australia (Orcid Id: 0000-0001-6832-4173) Twitter: @Louise_Hickson LinkedIn: https://www.linkedin.com/in/louise-hickson-ba273640/

Anne Forster - Leeds Institute of Health Sciences (LIHS), University of Leeds, Leeds, UK

Caroline Fairhurst- York Trials Unit, Department of Health Sciences, University of York, ARRC Building, York, UK (Orcid iD 0000-0003-0547-462X)

Catherine Hewitt- York Trials Unit, Department of Health Sciences, University of York, ARRC Building, York, UK (Orcid iD 0000-0002-0415-3536)

Rob Gardner -Audiology Department, Bradford Royal Infirmary, Bradford, UK

Kate Iley - Audiology Department, York Hospital, York, UK

For peer review only - http://bmjopen.bmj.com/site/about/guidelines.xhtml

Lorraine Gailey- Hearing Link, Eastbourne, UK

Nicholas J Thyer – Leeds Institute of Cardiovascular and Metabolic Medicine (LICAMM,) University of Leeds, Leeds, UK (Orcid iD 0000-0002-0025-783X)

Corresponding author: Dr Judith Watson: jude.watson@york.ac.uk

Total word count of main text= 5421

For peer review only

For peer review only - http://bmjopen.bmj.com/site/about/guidelines.xhtml

Abstract:

Objective: To establish the acceptability and feasibility of delivering the Active Communication Education (ACE) programme to increase quality-of-life through improving communication and hearing aid use in the UK National Health Service.

Design: Randomised controlled, open feasibility trial with embedded economic and process evaluations.

Setting: Audiology Departments in two hospitals in two UK cities.

Participants: Twelve hearing aid users aged 18 years or over who reported moderate or less than moderate benefit from their new hearing aid.

Interventions: Consenting participants (along with a significant other) were to be randomised by a remote, centralised randomisation service in groups to Active Communication Education (ACE) plus treatment-as-usual (intervention group) or treatment-as-usual only (control group).

Primary outcome measures: The primary outcomes were related to feasibility: recruitment, retention, treatment adherence and acceptability to participants, and fidelity of treatment delivery.

Secondary outcome measures: International Outcomes Inventory for Hearing Aids, Self-Assessment of Communication, EQ-5D-5L and Short-Form 36. Blinding of the participants and facilitator was not possible.

Results: Twelve hearing aid users and six significant others consented to take part. Eight hearing aid users were randomised: four to the intervention group; and four to treatment-as-usual only. Four significant others participated alongside the randomised participants. Recruitment to the study was very low and centres only screened 466 hearing aid users over the 15-month recruitment period, compared with the approximately 3,500 anticipated. Only one ACE group and

For peer review only - http://bmjopen.bmj.com/site/about/guidelines.xhtml

one control group were formed. ACE could be delivered and appeared acceptable to participants. We were unable to robustly assess attrition and attendance rates due to the low sample size.

Conclusions: Whilst ACE appeared acceptable to hearing aid users and feasible to deliver, it was not feasible to identify and recruit participants struggling with their hearing aids at the 3-month post-hearing aid fitting point.

Trial registration: ISRCTN28090877

Word count: 293

Keywords: Active Communication Education; Audiology; Feasibility; Hearing Aid; Randomised Controlled Trial; Quality of Life

Strengths and limitations of this study:

- This is the first study to explore the potential of using ACE as a tool to improve communication and hearing aid benefit for struggling adult hearing aid users and their significant others.
- This is the first study to identify and evaluate the processes needed to deliver an RCT of treatment-as-usual plus ACE versus treatment-as-usual alone within audiology clinics.
- Low recruitment meant some of the trial objectives were not robustly assessed.

For peer review only - http://bmjopen.bmj.com/site/about/guidelines.xhtml

INTRODUCTION

Age-related hearing impairment is reported as the third most common chronic condition affecting approximately 328 million (91%) middle and older aged adults worldwide[1] and 10 million adults in the United Kingdom (UK) [2,3] with the majority having mild or worse hearing impairment, which progressively deteriorates with age.[4] Hearing loss often impacts on quality-of-life and general well-being [5,6,7] such as increased occurrences of depression, [8] social isolation, [7] poor social interactions, [9,10,11] and cognitive dysfunction,[12] as well as increased risk of developing dementia.[13] In addition the impact on normally-hearing significant others (SOs) living with hearing-impaired people, is often overlooked where similar problems are reported.[9,14,15,16,17]

In spite of robust evidence that hearing aid use reduces the negative impact of age-related hearing impairment on quality-of-life,[5,18,19,20] it is estimated that up to 30% of UK adult hearing aid owners do not use them regularly or at all.[3,21,22,23] The reasons behind this are complex. [24] Numerous barriers and facilitators to successful hearing aid use have been identified as being related to expectations of benefit and meaningful participation in everyday life. [25,26]

The Active Communication Education (ACE) programme [27] trains participants to develop solutions to specific difficult communication scenarios that commonly lead to their avoidance of, or reduced participation in, daily activities. Three small studies have demonstrated benefits in improving communication function and hearing related quality-of-life when evaluated as an *alternative* to hearing aid fitting.[28,29,30] However, the effect of ACE on improving hearing aid benefit when delivered *in addition* to hearing aid fitting has not been evaluated. The ACE To HEAR

For peer review only - http://bmjopen.bmj.com/site/about/guidelines.xhtml

study (**A**ctive **C**ommunication **E**ducation **To** improve **HEAR**ing) aimed to assess whether a large randomised controlled trial (RCT) to evaluate the effectiveness and cost-effectiveness of ACE plus treatment-as-usual (TAU) versus TAU alone, was feasible within UK National Health Service (NHS) Audiology Departments.

DESIGN AND METHODS

The methods are summarised below and published in more detail elsewhere.[31] This was an open feasibility RCT with embedded economic and process evaluations. The required regulatory approvals were in place prior to study commencement.

Setting

The participating centres were York Hospital, York Teaching Hospitals NHS Foundation Trust, and Bradford Royal Infirmary, Bradford Teaching Hospitals NHS Foundation Trust.

Intervention

TAU was defined as a referral from a GP to audiology services to treat permanent hearing loss. It comprised of up to two appointments for hearing aid fitting and a third face-to-face or telephone follow-up appointment. The ACE to HEAR intervention consisted of five group sessions delivered as described in the published ACE manual [27] by a trained audiologist facilitator to groups of 5-7 hearing aid users and their SOs, where possible.

Study objectives

For peer review only - http://bmjopen.bmj.com/site/about/guidelines.xhtml

The study objectives were established around evaluating the delivery of the ACE intervention and the feasibility of trial delivery to assess:

- trial up-take rates, eligibility, and acceptability of clinic location;
- recruitment rates, the randomisation process and time to accrue ACE groups;
- ACE attendance and retention amongst participants randomised to the intervention group;
- acceptability of ACE with participants, SOs and audiologists;
- capability, capacity and willingness of audiology departments to support delivery of ACE within existing services;
- intervention fidelity of delivering ACE;
- the acceptability of study processes to participants, SOs and audiologists;
- patient reported outcome measures (PROMs) including piloting a bespoke resource use questionnaire and estimate parameters to be used when designing full-scale RCT.

Sample size and randomisation

Since this was a feasibility trial, no formal power calculation was conducted. We aimed to recruit 88 hearing aid (HA) users aged 18 years or over receiving TAU in the two participating centres, along with their SOs. The full eligibility criteria can be found in the published protocol.[31] The study sites estimated that there would be approximately 400 new hearing aids fitted across the study centres each month. Assuming 10% eligibility, 35% did not attend (DNA) and 30% consent rate, around 350 cases per month across the two centres would have needed to be screened to recruit the required 88 HA users in 12 months.

For peer review only - http://bmjopen.bmj.com/site/about/guidelines.xhtml

In the initial months of the trial (April to August 2017), eligibility criteria had included Q1 and Q2 from the International Outcomes Inventory for Hearing Aids (IOI-HA). [32] Q1 required self-reported HA use of less than three hours a day and Q2 required *less than moderate* benefit for eligibility. However, early scrutiny of the Q1 recruitment data showed evidence of over reported hearing aid use, including a variable presence of confirmatory HA log data and it was therefore removed as a criterion. Q2 was also reviewed and considered too stringent; eligibility was changed to include *moderate* as well as *less than moderate benefit* as these HA users were also considered to be struggling. These changes were implemented as of 29th August 2017.

Eligible, consenting HA users from the same study site who had completed baseline assessments were to be randomised 1:1 by a remote, centralised randomisation service (provided by York Trials Unit) in batches of 10–14 using block randomisation in a single large block per batch. This was to allow groups of 5-7 to be formed for the ACE intervention sessions. The target was to randomise participants within three weeks of consent.

Measurements

Data collected from participants at baseline via self-completed questionnaires were: IOI-HA;[32] Self-Assessment of Communication (SAC);[33] EQ-5D-5L;[34,35] and Short-Form 36 (SF-36)[36], resource use and demographics. SOs completed baseline questionnaires collecting: demographics; IOI-HA: version for Significant Others (IOI-HA-SO);[37] and Significant Other Scale for Hearing Disability (SOS-HEAR).[14]

For peer review only - http://bmjopen.bmj.com/site/about/guidelines.xhtml

Those in the ACE group also completed a questionnaire at the end of ACE session five. Measures were IOI-HA and the International Outcomes Inventory for Alternative Interventions (IOI-AI).[38] Their SOs completed IOI-HA-SO, SOS-HEAR, and the International Outcome Inventory for Alternative Interventions: version for Significant Others (IOI-AI-SO).[37]

The TAU participants were mailed questionnaires containing the IOI-HA at the equivalent time point; with their SOs completed IOI-HA-SO and SOS-HEAR. Participants and their SOs were asked additional questions regarding their thoughts on the programme (ACE arm only) and being approached to take part in the study (both arms). The facilitator collected data during all five ACE sessions in relation to participants' attendance at sessions, SO attendance and their relationship to participant.

At three months post-randomisation both groups (ACE and TAU) and their SOs completed the same measures as at baseline, and those in the ACE group and their SOs also completed the IOI-AI and associated IOI-AI-SO.

The feasibility and the potential for a future large-scale study was to be determined by a set of stop: go criteria (Table 1).

Table 1: Stop: go criteria

Criteria	Measure
Seventy per cent of recruitment targets attained for all research components	Numbers referred; numbers struggling with their hearing aid; number of exclusions.
Study consent/retention rates and proposed sample sizes, indicate delivery of the full RCT is plausible within a 5-year study period	Numbers screening; numbers consenting; numbers who declined to participate and

For peer review only - http://bmjopen.bmj.com/site/about/guidelines.xhtml

	reasons for declining; numbers withdrawing.
Ninety per cent of ACE groups of five to seven consented participants formed within the intervention window with participants attending three of five sessions	Time taken to recruit and logistics of recruiting an optimally sized and located ACE group; time ACE started after randomisation (ACE intervention window). Number of consented participants who failed to attend ACE sessions; number who missed ACE intervention window (i.e. unable to attend an ACE group within 1-3 weeks after randomisation).
Economic, acceptability, outcome measure and fidelity evaluation data successfully collect	Amount of missing data, although measures with over 10% missing data may be modified/replaced prior to the main trial.
Participants, SOs and audiologists evaluate acceptability of the ACE and RCT positively	Number given an appointment for an ACE group session; number of consented participants who failed to attend ACE sessions; Facilitator's adherence to the ACE protocol (fidelity); participant and SO thoughts regarding the study (interviews)

Statistical analysis

A single analysis was conducted at the end of the trial and undertaken in Stata v15. Baseline and outcome data for HA users are summarised overall and by randomised group, and are reported as mean, standard deviation (SD), median, minimum and maximum for continuous data, and counts and percentages for categorical data. The amount of missing data for each variable is also reported. The recruitment rate is summarised by month, overall, and by site. Attendance at the ACE sessions by participants and their SOs are detailed.

Questionnaire return rates at each time point are presented overall and by group. It was planned that SDs would be presented for PROMs with 80% confidence intervals to inform future sample size

For peer review only - http://bmjopen.bmj.com/site/about/guidelines.xhtml

calculations. An analysis to investigate sensitivity of the measures to change, by calculating the standardised response mean, was also planned.

The number of participants withdrawing from the ACE intervention and/or the trial and any reasons for withdrawal are summarised.

Economic analysis and quality of life data

The present trial aimed to determine the feasibility of conducting an economic evaluation of the ACE to HEAR intervention taking a broad perspective accounting not only for NHS costs but also those observed by HA users. In the UK, NICE advocates the use of the EQ-5D for measuring quality-of-life; however, the EQ-5D is not always the most sensitive tool for particular populations for whom the majority of its dimensions may not apply.[39] In a United States analysis of a similar population, the SF-36 was used to generate QALYs rather than the EQ-5D, with the SF-36 showing a reduction in the cost per QALY in favour of the intervention.[28] Given the UK preference for the EQ-5D, and the fact that it is shorter, thus less onerous to complete, participants in the feasibility trial were asked to complete both instruments to allow for comparison. Completion rates and mean scores for both instruments are summarised.

A bespoke resource use questionnaire was also designed to estimate the potential resource implications of the intervention versus usual care. Completion rates and mean resource use by item are summarised.

Process evaluation

For peer review only - http://bmjopen.bmj.com/site/about/guidelines.xhtml

For peer review only

The number of ACE sessions delivered was recorded and, to assess fidelity of delivering ACE, the facilitator completed a self-monitoring form, scoring adherence to the ACE manual for each session.

Following delivery of all the sessions, the facilitator was interviewed to explore the training and implementation process and their experiences of delivering ACE including barriers/facilitators to adhering to the ACE protocol. In addition, three audiologists were interviewed, exploring the capability, capacity and willingness of their audiology departments to support the ACE study within their existing services. The acceptability of study processes were also explored.

Three participants in the ACE intervention group, plus one SO, were interviewed after the completion of the ACE sessions. The semi-structured interviews explored the acceptability of the ACE (e.g. venue, timing, content), its perceived impact (reflecting on HA outcomes) as well as exploration of participant's views on study processes (e.g. recruitment, outcome measures and timing).

Interviews were audio-recorded, transcribed verbatim and analysed (with NVivo-11), using the Framework approach,[40] and then interpreted by team members.

Patient and Public Involvement

The development of this research study was informed by a variety of patient and public involvement (PPI) activities. A funded public engagement event about public perceptions of hearing impairment helped inform the research question as participants identified a need for wider availability of treatments in addition to hearing aids especially non-technological interventions, such as communication education. This feedback led to the choice of an interactive communication-based intervention rather than an

For peer review only - http://bmjopen.bmj.com/site/about/guidelines.xhtml

informational one and reinforced the need to ensure that routine practical information about hearing aids and hearing impairment is delivered consistently and checked after fitting. A focus group was then held to consult with service users on the proposed research question, study design and intervention delivery including study information and consent procedures, barriers and facilitators to participation, communication scenarios and motivating factors to becoming more active communicators. In addition, the charity Hearing Link, who have extensive experience of PPI and managing and delivering group interventions of this type, were consulted about involving public and patients in operationalising and delivering ACE. The formation of a Project Advisory Panel (PAP) consisting of service users ensured PPI involvement continued during the conduct of this study. Patients were not directly involved in recruitment to the study. A lay summary of the findings will be sent to the participants as well as disseminated via patient forums, Trust bulletins and public interest groups.

RESULTS

Recruitment

The flow of participants through the trial is shown in a CONSORT diagram (Figure 1). Screening and recruitment occurred from 01st April 2017 to the end of June 2018; 466 HA users were screened whilst attending a follow-up HA fitting appointment at Bradford (n=275) or contacted by telephone 3-months post-fitting at York (n=191). Of these, 86 (18.5%) were eligible but 74 declined to participate. Twelve participants were recruited into the trial (14.0% of those eligible); one from Bradford and eleven from York (Figure A in supplementary material). Reasons for ineligibility (n=380) and non-consent (n=74) can be found in Figure 1. The most common reason was that the HA helped too much (81.8% of n=380 excluded). Six SOs also consented to take part.

For peer review only - http://bmjopen.bmj.com/site/about/guidelines.xhtml

It was found that 22.6% of HA users who were screened (n=380) were eligible (based on the applicable screening criteria at the time of screening; additional participants were identified as 'would have been eligible' when the inclusion criteria were changed); however, on average only 33 HA users were screened each month, and this was confounded by the 13.9% consent rate (% of the 86 eligible, 8.9% when including those who would have been eligible under the new criteria).

Randomisation

Randomisation was intended to be performed in batches of 10-14 participants. However, due to slow recruitment, the decision was made in January 2018 to randomise the participants recruited from York thus far as a smaller block to allow at least one ACE group to be run. The randomisation was undertaken as a block of eight, four participants to each group. Additionally, four SOs were also recruited, and allocated to their respective participant's group – three to TAU and one to the intervention group. There were insufficient numbers of participants to randomise the four subsequently recruited participants, and the two SOs.

The average time from screening to randomisation was 19.5 weeks (range 10.6 to 33.6 weeks) and the target three-week window for randomisation post-consent was missed for all participants. If the group had been randomised immediately after the eighth participant was consented (randomisation was delayed in the hope of a larger group), then the average time would have been 8.9 weeks, with 37.5% meeting the three-week target. The first ACE session was conducted 11 days after randomisation.

Participants

For peer review only - http://bmjopen.bmj.com/site/about/guidelines.xhtml

For peer review only

The baseline characteristics of randomised participants and their SOs can be found in Table 2. As there was only one SO in the intervention group, data are only presented overall to prevent disclosure.

Table A in the supplementary material shows the screening data on use and benefit of HA, threshold data and hearing loss aetiology for the randomised participants by group and overall.

Table 2: Baseline characteristics for the randomised participants, by group and overall and overall for the SOs

	**ACE Intervention participants (n=4)**	**TAU participants (n=4)**	**All participants (n=8)**	**All SOs (n=4)**
**Age, years**				
Mean (SD)	73.0 (7.8)	73.3 (6.0)	73.1 (6.4)	73.6 (7.3)
Median (min, max)	72.9 (64.1, 82.1)	73.9 (65.9, 79.5)	73.6 (64.1, 82.1)	76.6 (62.9, 78.3)
**Gender, n (%)**				
Male	3 (75.0)	2 (50.0)	5 (62.5)	2 (50.0)
Female	1 (25.0)	2 (50.0)	3 (37.5)	2 (50.0)
**Length of time concerned about hearing, years**				N/A
Mean (SD)	21.3 (7.5)	3.5 (1.9)	12.4 (10.8)	
Median (min, max)	20 (15, 30)	4 (1, 5)	10 (1, 30)	
**Ethnicity, n (%)**				
White British	4 (100.0)	4 (100.0)	8 (100.0)	4 (100.0)
Other	0 (0.0)	0 (0.0)	0 (0.0)	0 (0.0)
**Marital Status, n (%)**				
Married/Civil partner	2 (50.0)	3 (75.0)	5 (62.5)	4 (100.0)
Separated	0 (0.0)	0 (0.0)	0 (0.0)	0 (0.0)
Divorced	1 (25.0)	1 (25.0)	2 (25.0)	0 (0.0)
Missing	1 (25.0)	0 (0.0)	1 (12.5)	0 (0.0)
**Living arrangements, n (%)**				
Owned outright	3 (75.0)	4 (100.0)	7 (87.0)	4 (100.0)

For peer review only - http://bmjopen.bmj.com/site/about/guidelines.xhtml

Owned with	0 (0.0)	0 (0.0)	0 (0.0)	0 (0.0)
mortgage	0 (0.0)	0 (0.0)	0 (0.0)	0 (0.0)
Rented	1 (25.0)	0 (0.0)	1 (12.5)	0 (0.0)
Privately rented				
**Main activity, n (%)**				
Full time employment	0 (0.0)	0 (0.0)	0 (0.0)	0 (0.0)
Part time	0 (0.0)	0 (0.0)	0 (0.0)	0 (0.0)
Self-employed	0 (0.0)	0 (0.0)	0 (0.0)	0 (0.0)
Unable to work due to health	1 (25.0)	0 (0.0)	1 (12.5)	0 (0.0)
Retired	2 (50.0)	4 (100.0)	6 (75.0)	4 (100.0)
Student	0 (0.0)	0 (0.0)	0 (0.0)	0 (0.0)
Retired & self-employed	1 (25.0)	0 (0.0)	1 (12.5)	0 (0.0)
**Qualifications, n (%)**				
No formal qualifications	2 (50.0)	1 (25.0)	3 (37.5)	0 (0.0)
GCSEs	1 (25.0)	1 (25.0)	2 (25.0)	1 (25.0)
A/AS Levels	0 (0.0)	0 (0.0)	0 (0.0)	0 (0.0)
Higher education	0 (0.0)	1 (25.0)	1 (12.5)	2 (50.0)
Further higher education	0 (0.0)	1 (25.0)	1 (12.5)	0 (0.0)
Vocational	0 (0.0)	0 (0.0)	0 (0.0)	0 (0.0)
Other	1 (25.0)	0 (0.0)	1 (12.5)	1 (12.5)

For peer review only

Participants had mild to moderately severe high frequency sensorineural hearing loss, except for one who reported an additional conductive element to their hearing loss. Five participants reported using their HA for less than 4 hours a day and three for more than 8 hours a day. All participants reported less than moderate levels of benefit. Those randomised to the ACE intervention reported that they had been concerned about their hearing for markedly more years than those allocated to TAU.

Intervention attendance

For peer review only - http://bmjopen.bmj.com/site/about/guidelines.xhtml

Of the four York participants who were randomised to receive the intervention, one attended all five sessions, one attended four sessions, one three sessions, and one participant did not attend any of the five sessions. This equates to an average attendance rate of 60%. Of these four participants, only one had a SO who wished to participate, and they attended all the sessions that their associated participant attended.

Follow-up and standardised measures

At the end of the sessions (approximately week 5), 7/8 participants (87.5%) returned the postal questionnaire (ACE 4/4 (100%); TAU 3/4 (75%)). In addition, 3/4 (75%) SOs returned their questionnaires (ACE 1/1 (100%); TAU 2/3 (67%)).

At 3-months post-randomisation, the retention rate was 75%; three out of four participants from each group returned the questionnaires. Three of the four (75%) SOs returned their 3-month post-randomisation questionnaires. No participants withdrew in this study (but one participant in the ACE group did not attend a single session).

The results from Tables B – D (supplementary material), completed by the participants at both baseline and at least one of the follow-up time points, show that the measures used were well completed. The average IOI-HA score at all three time points was in the mid-to-high range, indicating average hearing outcomes; there was no obvious change in this result in either group over time. There does appear to be imbalance in the SAC between TAU and ACE participants, with those in the control group rating themselves as being worse at communicating. However, as with the IOI-HA there appears to be no change in the average score across the follow-up period.

For peer review only - http://bmjopen.bmj.com/site/about/guidelines.xhtml

The outcome measures for the SOs were equally well completed (Tables E & F: supplementary material). Since only one SO completed the IOI-AI-SO, the results are not presented.

Out of all of the returned questionnaires (n=8, 6 and 6 for participants and n=4, 3 and 3 for SOs at baseline, W5 and M6 respectively) there was only one instance where a PROM was not completed sufficiently to allow for scoring (IOI-AI for an ACE participant at M6).

The 80% confidence intervals for SDs for PROMs were not calculated, and the planned analysis to investigate the sensitivity of the measures to change was not conducted as it was considered they would not produce reliable results due to the low sample size.

Health Economics

Of the eight participants at baseline, all completed the EQ-5D-5L in full, compared to six full completions (75%) and two partial completions for the SF-36 (25%). At 3-months post-randomisation, two participants (one from each group) did not complete the questionnaire; all remaining participants completed both the EQ-5D-5L and SF-36 in full. At baseline, participants allocated to the intervention group had a slightly higher mean score compared to participants in the TAU group (mean difference 0.057) (Table 3). This difference was reversed at 3 months with participants allocated to the TAU group having a slightly higher mean score (mean difference -0.186); although as the sample size is small, no conclusions can be drawn from this.

Table 3: EQ-5D-5L mean (SD) scores and mean difference at baseline and 3 months post-randomisation follow-up

For peer review only - http://bmjopen.bmj.com/site/about/guidelines.xhtml

Follow-up	ACE Mean (SD)	TAU Mean (SD)	Mean Difference (ACE-TAU)
Baseline	0.670 (0.333)	0.613 (0.512)	0.057
3 Months post-randomisation	0.686 (0.411)	0.871 (0.133)	-0.186

These findings were also reflected in the results of the SF-36 (Table 4). At baseline, participants allocated to the intervention group scored on average higher on dimensions of physical role, general health, social function, emotional role and mental health as well as on the overall physical and mental components. At the 3-month post-randomisation follow-up, participants allocated to the intervention group only scored higher on the measure of vitality.

Table 4: SF-36 mean (SD) scores and mean difference at baseline and 3 months post-randomisation follow-up

SF-36 Score		ACE Mean (SD)	TAU Mean (SD)	Mean difference (ACE - TAU)
**Baseline**				
	Physical functioning	61.67 (28.77)	66.25 (44.60)	-4.58
	Physical role	54.69 (40.63)	48.44 (38.32)	6.25
	Bodily pain	59 (32.19)	65.33 (46.88)	-6.33
	General health	61.5 (36.30)	49.75 (24.31)	11.75
	Vitality	45.31 (22.46)	52.08 (15.73)	-6.77
	Social functioning	68.75 (33.07)	58.33 (50.52)	10.42
	Emotional role	77.08 (26.68)	77.08 (15.77)	0
	Mental health	68.75 (23.94)	63.33 (12.58)	5.42
	Overall physical component	41.11 (14.69)	39.44 (24.22)	1.67
	Overall mental component	46.87 (10.77)	45.86 (2.31)	1.01
**3 Months post-randomisation**				

For peer review only - http://bmjopen.bmj.com/site/about/guidelines.xhtml

Physical functioning	45 (39.05)	83.33 (10.41)	-38.33
Physical role	41.67 (38.19)	68.75 (22.53)	-27.08
Bodily pain	66.67 (57.74)	70 (6.93)	-3.33
General health	58 (41.87)	58.67 (15.28)	-0.67
Vitality	54.17 (36.62)	52.08 (3.61)	2.08
Social functioning	62.5 (45.07)	87.5 (21.65)	-25
Emotional role	75 (25.0)	88.89 (19.25)	-13.89
Mental health	78.33 (20.21)	78.33 (16.07)	0
Overall physical component	36.10 (21.08)	45.84 (7.134)	-9.74
Overall mental component	51.33 (10.23)	51.67 (11.97)	-0.34

The resource use questionnaire was not well completed, with one full completion (12.5%) and five partial completions (62.5%) at baseline and six partial completions only at 3 months post-randomisation (75%). There was no difference in completion by trial group. Overall, very low levels of resource use were reported across both groups with most resource use centred on hearing health care professionals (Tables G and H: supplementary material).

Appropriate unit costs to be applied for each resource use type were identified through local costings and national databases including PSSRU [41] and NHS reference costs.[42]

Adverse events

None were recorded.

Process evaluation

Five ACE sessions were delivered. At the first session, (1) introduction to ACE and communication needs analysis, the group decided which modules they wanted to use, since each addressed a

For peer review only - http://bmjopen.bmj.com/site/about/guidelines.xhtml

different aspect of communication. They chose: conversation in background noise (session 2); conversation around the house (session 3); communication with difficult speakers (session 4); and public-address systems (session 5). Three HA users and one significant other attended.

Facilitator views and fidelity

Overall, the facilitator considered the ACE programme to have had a positive impact because some of the content was new knowledge for participants, and a few had reported implementing some communication strategies with success.

"The information I was able to give I think was really positive and helped them a lot. I think having spoken to them the following week they'd been able to implement some of them (communication strategies) and found those quite useful. Particularly things like watching the TV at home, that they could put the subtitles on or could change the sound settings or that kind of thing. I think they all felt a bit more empowered by the end of it, that they were able to admit that they had a hearing loss and admit that things weren't going to be perfect in every situation."

Key challenges to its delivery were low numbers of participants which limited discussion, some reluctance amongst participants to "bother others" to help with communication, and participants wanting to discuss hearing aid technology and their frustrations with audiology services (instead of communication). As the sessions progressed, the facilitator became more flexible in delivering the programme, ensuring that these other topics were covered. This was reflected in the fidelity scores which changed from "adherence to the protocol" all the time (session 1), most of the time (sessions 2 and 3) and sometimes (sessions 4 and 5).

Participants' views

For peer review only - http://bmjopen.bmj.com/site/about/guidelines.xhtml

In reflecting on the ACE programme, two participants and one SO gave several examples of how it was helpful. They had tried out different strategies: sitting with their back to the wall, adjusting settings on their TV, using subtitles and checking if the loop system is switched on when visiting theatres. A key change was increased confidence in speaking out when they cannot hear and asking others to help with this, for example; facing them when in conversation, requesting people to repeat themselves rather than speak more loudly and closing a window to reduce background noise.

"It also brought it home to me how important it is to tell other people that you've got a hearing problem, because you're reluctant to do it when you're first deaf. And so you muddle along and you say "yes, yes", and then sometimes you know that they're getting annoyed so then you pretend to hear what was said and of course you laugh in the wrong place or you can't join in the jokes. I always miss punchlines. So, I learned that you must say to people "I'm sorry, I can't hear, can I sit here? Can we do this? Can we turn that off?"

That said, there was still some reluctance to ask others for help, with participants favouring strategies that they could do themselves. One participant said he felt the training "wasn't right for me" and that the sessions had not changed anything for him.

Audiologists' views

The audiologists said they had been able to deliver the study within their departments; adapting their routine follow-up process to include the ACE screening questionnaire. However, the study was more time consuming than anticipated and requiring extra support from the university team to help answer questions from HA users was mentioned in one department. Suggested reasons for the failure to recruit were: too strict eligibility criteria (which were changed); a reluctance of older people to participate in research; communication barriers are already discussed in routine appointments; and many HA users nowadays are younger and want to focus on the benefits that

For peer review only - http://bmjopen.bmj.com/site/about/guidelines.xhtml

technology afford. Ideas for improving recruitment were: to recruit people at their initial fitting, or people who are long term HA users; offer more days and locations for follow-up appointments so more people attend; present ACE as a support group rather than a research study; and send flyers out or invite potential participants in for a coffee morning to discuss the study.

Finally, in discussing trial processes, the facilitator and participants observed it had taken a long time to set up the first ACE group and that the information needed to be clearer that the focus of the programme was communication. Participants and audiologists mentioned the length and complexity of the ACE questionnaires. It was difficult to deliver these over the telephone with some elderly HA users.

DISCUSSION

This is the first study to explore the potential of ACE as a tool to improve communication and hearing aid benefit for struggling adult hearing aid users and their significant others. It is also the first study to identify and evaluate the processes needed to deliver an RCT of ACE within the general practitioner direct referral pathway for new NHS hearing aid users in the UK (TAU). The key findings from this feasibility study are: (1) there is some (limited data) to suggest the intervention is potentially acceptable and feasible to HA users, SOs and audiologists; (2) the data show that the data collection, monitoring and reporting aspects of the trial delivery were feasible and acceptable; finally, (3) we were unable to demonstrate the efficacy of recruitment to the trial within a NHS setting. We did, however, identify some problems that underpin the difficulty of recruitment in this context.

For peer review only - http://bmjopen.bmj.com/site/about/guidelines.xhtml

Recruitment to RCTs is challenging and it is important to report the root causes of recruitment difficulties so that mistakes are not repeated in future studies. Emerging data was explored by the trial team to consider how to problem solve and optimise recruitment and associated procedures.

The number of patients fitted with a HA who were followed-up and screened for eligibility (n=466), was extremely low in comparison to the number of expected HA fittings (>4000). The study sites' data reporting mechanisms struggled to accurately report the patient flow (monthly HA fittings) through this pathway but actual non-attendance rates for patients offered a follow-up appointment were calculated to be 30%, the level planned for, and do not account for this short fall.

During the study planning phase, both sites agreed to introduce face-to-face or telephone follow-up (not previously offered, despite NICE guidelines indicating this as good practice [43]) for all patients in the TAU pathway. The low number of patients seen for follow-up and then screened for eligibility by both sites suggests fully embedding and scaling-up this change took longer in practice than anticipated. Not all patients in the pathway were offered post-fitting follow-up and not all who attended were screened, leading to a significantly smaller pool of potential participants to screen (problems also highlighted by Donovan et al. [44]).

There is some evidence that RCTs with more restrictive eligibility criteria exhibit poorer recruitment than those with wider criteria.[45] After 6 weeks of missed monthly recruitment targets, we reviewed our eligibility criteria. As noted previously, the relationship between daily hours of HA use and HA outcomes is not well specified.[46] Consequently, criterion Q1 (hours of use), was dropped

For peer review only - http://bmjopen.bmj.com/site/about/guidelines.xhtml

and the level of benefit (Q2) criterion widened to include patients reporting moderate benefit. Audiologists' feedback supported these changes and by the end of recruitment over 26% of those screened had identified as struggling. We had factored in a conservative estimate of 10%, based on clinical experience at each study site. The disparity reflects the modified eligibility criteria and variability in self-reported HA use data noted earlier.

Despite these problems, the screening process appeared to successfully identify the target population since the demographic profile of those screened and consented was as expected. The range of ages (64.1 – 82.1) indicated that it was older, not younger participants who were both eligible and willing to participant. Their hearing profile was consistent with that expected from struggling hearing aid users.

Our a priori estimate (30%) of willing participants was closer to 14%. Since willingness is dependent on project specifics, population demographics, and patient perceptions of the research's value and worth, this was a feasibility parameter of interest. We could find no relevant study reporting detailed recruitment statistics in this context for comparison.

Comparing our recruitment issues with earlier ACE studies [27,29] is difficult since their context was very different, not least because they recruited from an already motivated population, rather than a specific publicly funded pathway known to foster motivational challenges, as reported here. Descriptions of the burden associated with RCT participation include, anxiety about taking part in research, factors related to processing trial information, demanding follow-up, and lack of appropriate post-trial management.[47] Our patients were possibly already feeling overwhelmed

For peer review only - http://bmjopen.bmj.com/site/about/guidelines.xhtml

by managing their new HA and were less motivated to engage in research. Of the eligible, non-consenting patients, the majority gave no reason, were not interested or were too busy. Hickson et al [28] reported a much lower non-consent rate (20% compared to 86% here) and none of the burdens identified by Naidoo and colleagues [47] were overtly apparent.

It is notable that patients' decisions to not take part were made at clinical follow-up; few were passed on to the Research Nurse for further information and consent. Audiologists reported struggling to find time to answer participants' questions fully (either by phone or face-to-face). Thus, the opportunity for prospective participants to process the trial information at this point may not have been optimum, and they declined referral to the Research Nurse. However, this was not reflected in the patients' narrative on non-consenting.

Research involvement can also be burdensome for clinicians and time is a frequently voiced barrier to their participation in research.[48,49] Pressures from usual clinical practice, [48,50] time demands of recruitment and follow-up, leading to increases in workload pressure [51] are associated with under-recruitment, as is a lack of research experience, [48,52] which was something the interviews with the screening audiologists revealed as a critical concern.

During the project development, audiologists and service managers said they could adapt their practice to include ACE screening. However, over time, they considered the screening questionnaire to be onerous and one site felt that they required more help from the "University Team" to answer patient's questions, despite having a dedicated research nurse/practitioner on site.

For peer review only - http://bmjopen.bmj.com/site/about/guidelines.xhtml

It is unlikely that patients declined to participate because they thought the research unimportant since participant interviews and pre-study PPI work indicated our research question was perceived as important.

Important aspects of recruitment such as ensuring simple procedures for information provision and consenting, and that study-related follow-ups do not increase pressure on usual clinical follow-ups (either for clinicians or patients) [52,53] were not really tested here because so few participants consented. Interviews with those who did indicated these aspects were not off-putting.

Many aspects of the study processes worked well. The randomisation process was viewed as acceptable by the small cohort recruited although insufficient numbers were achieved to allocate the last four participants recruited. There was no sense from the recruitment process that SO recruitment was problematic or that including SOs had a detrimental effect on recruitment. The SO/participant ratio (albeit from minimal data) is commensurate with similar studies [15,28] and was viewed as a positive incentive by participants. Having a SO was not an inclusion criterion, not disadvantaging those without a SO.

The trial processes undertaken for collecting outcome measures worked well. Outcome measure completion rates and interview data suggests that processes worked and are acceptable to participants. Due to insufficient participant numbers, estimation of SD for the PROMS, quality of life tools and healthcare resource use questionnaires was not possible. Completion and retention rates both indicate that whilst we were unable to recruit into the trial, those who were randomised stayed involved, suggesting a trial may have been possible had recruitment not been an issue. Attempts to

For peer review only - http://bmjopen.bmj.com/site/about/guidelines.xhtml

increase recruitment via amendments to streamline recruitment paperwork and processes failed, and further study site training proved unsuccessful. We also planned to (1) add additional study sites; (2) change the point in the referral pathway at which HA users were approached, to the fitting appointment, rather than the 3-month follow-up appointment; and (3) to transfer all responsibility for recruitment from the NHS audiologists to the University research team. Ethical approval was gained for these adjustments and the funder granted the additional time required, but refused additional funding.

The ACE delivery processes appeared to work well, with the facilitator becoming more flexible over the five sessions to accommodate participant's needs within the ACE protocol. Participants mostly completed the programme and were generally positive in their feedback, citing changes they were making. This is in line with previous studies using ACE in Australia, Sweden and Chile.[28,30,54] All previous studies reported good adherence to the ACE program (>90% completed 3 or more of the 5 sessions), and positive outcomes were indicated in terms of communication. All studies found pre-post ACE improvements in communication strategy use, particularly for those with mild hearing loss; activity and participation and psychosocial well-being, with findings reflected in positive feedback. Despite the small numbers of participants in this current study, participants also reported positive benefits from completing the ACE programme. A key challenge to the efficacy of delivering ACE here was the small group sizes that limited group discussion, so it was interesting and encouraging to note the broad changes people made in their communication strategies because the ACE programme.

CONCLUSION

For peer review only - http://bmjopen.bmj.com/site/about/guidelines.xhtml

A future RCT using the recruitment strategy reported here is not likely to be feasible. The fundamental problem was in accessing sufficient struggling hearing aid users at follow-up. Recruiting hearing aid users when first fitted may be a better strategy for future research. Additional work to investigate which point in the pathway patients should best be approached and to identify the resources needed for good recruitment is required. Work to develop a robust service delivery model of follow-up for new adult HA users based on NICE guidelines will be crucial, as would implementing organisational support for NHS audiologists involved in research.

Acknowledgments

This work uses data provided by patients and collected by the NHS as part of their care and support and would not have been possible without access to this data. The Authors would also like to thank the members of the Project Advisory Panel (PAP), Emmanuelle Blondiaux-Ding, Philip Le Mare and Shona Hudson for their invaluable advice on many aspects of the project.

Contributors

NJT led on the original conception and design of the study with substantial contributions to the protocol from JW, CJ, EC, AF, LH, KB, CF, CH, CM, LG, RG and KI. As Chief Investigator, NJT takes overall responsibility for the work. CM liaised with the participants, delivered the ACE intervention and oversaw the PAP meetings. JW made substantial contributions to the trial design and management and manuscript drafting. EC was responsible for data analysis and data interpretation. KB was responsible for the health economic design and analysis. CF and CH provided further statistical advice and expertise on the study. CJ was responsible for qualitative interviews and data collection and transcript analysis. AF was involved in all aspects of the study

For peer review only - http://bmjopen.bmj.com/site/about/guidelines.xhtml

and KI and RG were recruiting site leads, responsible for aspects specific to Trust's service delivery and providing expert clinical support. All authors contributed to draft manuscripts, read and approved the final manuscript.

Approvals obtained

Ethical approval was granted by South East Coast-Surrey Research Ethics Committee (16/LO/2012) on 02nd December 2016 and Health Research Authority approval was obtained on 16th January 2017.

Funding Statement

This paper presents independent research funded by the National Institute for Health Research (NIHR) under its Research for Patient Benefit (RfPB) Programme (Grant Reference Number PB-PG-0215-36147). The views expressed are those of the author(s) and not necessarily those of the NHS, the NIHR or the Department of Health and Social Care.

Patient consent for publication

Not required

Competing Interests

None

Data Availability Statement

The data that support the findings of this study are available from the corresponding author, [JW], upon reasonable request.

For peer review only - http://bmjopen.bmj.com/site/about/guidelines.xhtml

Figure Legend

Figure 1: The flow of participants through the trial shown in a CONSORT diagram

REFERENCES

1. Organisation WH. WHO global estimates on prevalence of hearing loss: Mortality and Burden of Diseases and Prevention of Blindness and Deafness. Secondary WHO global estimates on prevalence of hearing loss: Mortality and Burden of Diseases and Prevention of Blindness and Deafness. 2012. http://www.who.int/pbd/deafness/WHO_GE_HL.pdf.

2. Davis AC. The Prevalence of Hearing Impairment and Reported Hearing Disability Among Adults in Great Britain. *Int J of Epidemiol* 1989;18(4):911-17.

3. AoHL. Hearing Matters: Taking action on hearing loss in the 21st century. http://www.actiononhearingloss.org.uk/supporting-you/policy-research-and-influencing/research/hearing-matters.aspx: Action on Hearing Loss, 2011.

4. Roth TN, Hanebuth D, Probst R. Prevalence of age-related hearing loss in Europe: a review. *Eur Arch Otorhinolaryngol* 2011;268(8):1101-07.

5. Bess FH, Lichtenstein MJ, Logan SA, et al. Hearing impairment as a determinant of function in the elderly. *J Am Geriatr Soc* 1989;37(11):123-28.

6. Chia EM, Wang JJ, Rochtchina E, et al. Hearing impairment and health-related quality of life: The blue mountains hearing study. *Ear Hear* 2007;28:187-95.

7. Health Quality Ontario. Social isolation in community-dwelling seniors: an evidence-based analysis. *Ont Health Technol Assess Ser* 2008;8(5):1-49.

8. Pronk M, Deeg DJH, Smits C, et al. Prospective effects of hearing status on loneliness and depression in older persons: Identification of subgroups. *Int J Audiol* 2011, 50(12): 887-896.

9. Hetu R, Jones L, Getty L. The impact of acquired hearing impairment on intimate-relationships - implications for rehabilitation. *Audiology* 1993;32(6):363-81.

10. Heine C, Browning C. The communication and psychosocial perceptions of older adults with sensory loss: a qualitative study. *Ageing Soc* 2004;24:113-30.

11. Heine C, Browning CJ. Communication and psychosocial consequences of sensory loss in older adults: overview and rehabilitation directions. *Disabil Rehabil* 2002;24(15):763-73.

12. Humes LE, Dubno JR, Gordon-Salant S, et al. Central Presbycusis: A Review and Evaluation of the Evidence. *J Am Acad Audiol* 2012;23(8):635-66.

13. Stephens D, Kramer SE. Living with hearing loss: The Process of Enablement. London: Wiley, 2009.

For peer review only

For peer review only - http://bmjopen.bmj.com/site/about/guidelines.xhtml

14. Scarinci N, Worrall L, Hickson L. The effect of hearing impairment in older people on the spouse: Development and psychometric testing of The Significant Other Scale for Hearing Disability (SOS-HEAR). *Int J Audiol* 2009;48(10):671-83.
15. Scarinci N, Worrall L, Hickson L. Factors Associated With Third-Party Disability in Spouses of Older People With Hearing Impairment. *Ear Hear* 2012;33(6):698-708.
16. Wallhagen MI, Strawbridge WJ, Shema SJ, et al. Impact of self-assessed hearing loss on a spouse: A longitudinal analysis of couples. *J Gerontol B Psychol Sci Soc Sci* 2004;59(3):S190-S96.
17. Brooks DN, Hallam RS, Mellor PA. The effects on significant others of providing a hearing aid to the hearing-impaired partner. *Br J Audiol* 2001; 35: 165–171
18. Kochkin S. MarkeTrak VIII patients report improved quality of life with hearing aid usage. *Hearing J* 2011;64(6):25-32.
19. Laplante-Levesque A, Hickson L, Worrall L. Rehabilitation of Older Adults With Hearing Impairment: A Critical Review. *J Aging Health* 2010;22(2):143-53.
20. Ferguson MA, Kitterick PT, Chong LY, et al. Hearing aids for mild to moderate hearing loss in adults. *Cochrane Database Syst Rev* 2017(9).
21. Gianopoulos I, Stephens D, Davis A. Follow up of people fitted with hearing aids after adult hearing screening: the need for support after fitting. *BMJ* 2002;325.
22. Hougaard S, Ruf S. EuroTrak I: A consumer survey about hearing aids in Germany, France and the UK. First global comparative study of hearing, hearing loss, and hearing aids. The *Hearing Rev* 2011;18(2):12-28.
23. Stephens D, Lewis P, Davis A, et al. Hearing aid possession in the population: Lessons from a small country. *Audiology* 2001;40(2):104-11.
24. Ng JH-Y, Loke AY. Determinants of hearing aid adoption and use among the elderly: A systematic review. *Int J Audiol* 2015;54(5):291-300.
25. Gagne JP, Jennings MB, Southall K. The ICF: A Classification System and Conceptual Framework Ideal for Audiological Rehabilitation. *Perspectives on Aural Rehabilitation and its Instrumentation* 2009;16(1):8-14.
26. Lockey K, Jennings MB, Shaw L. Exploring hearing aid use in older women through narratives. *Int J Audiol* 2010;49(8):542-49.
27. Hickson L, Worrall L, Scarinci N, editors. *ACE: Active Communication Education.* University of Queensland, 2015.
28. Hickson L, Worrall L, Scarinci N. A randomized control trial evaluating the Active Communication Education program for older people with hearing impairment. *Ear Hear* 2007;28(2):212-30.
29. Oberg M, Bohn T, Larsson U. Short- and Long-Term Effects of the Modified Swedish Version of the Active Communication Education (ACE) Program for Adults with Hearing Loss. *J Am Acad Audiol* 2014;25(9):848-58.

For peer review only - http://bmjopen.bmj.com/site/about/guidelines.xhtml

30. Rivera S, Marcotti A, Fuente A, et al. Adaptation of the “Active Communication Education” program into Spanish for older adults with hearing loss. *Int J Audiol* 2020. doi:10.1080/14992027.2020.1740801

31. Thyer NJ, Watson J, Jackson C, et al. Randomised controlled feasibility trial of the Active Communication Education programme plus hearing aid provision versus hearing aid provision alone (ACE to HEAR): a study protocol. *BMJ Open* 2018;8:e021502. doi:10.1136/ bmjopen-2018-021502

32. Cox RM, Alexander GC. The International Outcome Inventory for Hearing Aids (IOI-HA): psychometric properties of the English version. *Int J Audiol* 2002;41(1):30-35.

33. Schow RL, Nerbonne MA. Communication screening profile - use with elderly clients. *Ear Hear* 1982;3(3):135-47.

34. Herdman M, Gudex C, Lloyd A, et al. Development and preliminary testing of the new five-level version of EQ-5D (EQ-5D-5L). Quality of Life Research. *Qual Life Res* 2011;20(10):1727-36.

35. Janssen M, Pickard A, Golicki D, et al. Measurement properties of the EQ-5D-5L compared to the EQ-5D-3L across eight patient groups: a multi-country study. *Qual Life Res* 2013;22(7):1717-27.

36. Ware J, Sherbourne C. The MOS 36-item short-form health survey (SF-36) 1: conceptual framework and item selection. *Med Care* 1992;30:473-83.

37. Bellg AJ, Resnick B, Minicucci DS, et al. Enhancing Treatment Fidelity in Health Behavior Change Studies: Best Practices and Recommendations From the NIH Behavior Change Consortium. *Health Psychol* 2004;23(5):441-51.

38. Noble W. Extending the IOI to significant others and to non-hearing aid-based interventions. *Int J Audiol* 2002;41(1):27-29.

39. National Institute of Health and Clinical Excellence [NICE]. Guide to the methods of technology appraisal (PMG9). London: NICE 2013.

40. Ritchie J, Lewis J. *Qualitative Research Practice*. London: Sage Publications, 2003.

41. Curtis L, Burns A (2018) Unit Costs of Health and Social Care 2018, Personal Social Services Research Unit, University of Kent, Canterbury. https://doi.org/10.22024/UniKent/01.02.70995

42. Department of Health. NHS Reference Costs 2017/18. London: NICE 2018.

43. National Institute of Health and Clinical Excellence [NICE]. Hearing loss in adults: assessment and management (NG98). London, 2018.

44. Donovan JL, Paramasivan S, de Salis I, *et al.* Clear obstacles and hidden challenges: understanding recruiter perspectives in six pragmatic randomised controlled trials. *Trials* **15,** 5 (2014). https://doi.org/10.1186/1745-6215-15-5

For peer review only

45. Briel M, Speich B, von Elm E et al. Comparison of randomized controlled trials discontinued or revised for poor recruitment and completed trials with the same research question: a matched qualitative study. *Trials* 2019; **20:**800.

46. Perez E, Edmonds BA. A Systematic Review of Studies Measuring and Reporting Hearing Aid Usage in Older Adults since 1999: A Descriptive Summary of Measurement Tools. *PLoS ONE* 2012; 7(3): e31831. https://doi.org/10.1371/journal.pone.0031831

47. Naidoo N, Nguyen VT, Ravaud P et al. The research burden of randomized controlled trial participation: a systematic thematic synthesis of qualitative evidence. *BMC Med* 2020; 18;6 https://doi.org/10.1186/s12916-019-1476-5

48. Bullen T, Maher K, Rosenberg JP et al. Establishing research in a palliative care clinical setting: Perceived barriers and implemented strategies. *App Nurs Res* 2014*; 27*: 78-83 https://doi.org/10.1016/j.apnr.2013.10.007

49. Harding KE, Porter J, Horne-Thompson A et al. Not enough time or a low priority? Barriers to evidence-based practice for allied health clinicians. *J Contin Educ Health Prof* 2014*;34*(4): 224-231. https://doi.org/10.1002/chp

50. Messner DA, Moloney R, Warriner AH, et al. Understanding practice-based research participation: The differing motivations of engaged vs. non-engaged clinicians in pragmatic clinical trials. *Contemp Clin Trials Commun* 2016; 4, 136-140. https://doi.org/10.1016/j.conctc.2016.08.003

51. Paget SP, Caldwell PHY, Murphy J, et al. (2017). Moving beyond 'not enough time': factors influencing paediatric clinicians' participation in research. *Intern Med J, 47*(3), 299-306. https://doi.org/10.1111/imj.13351

52. Isaksson E, Wester P, Laska AC *et al.* Identifying important barriers to recruitment of patients in randomised clinical studies using a questionnaire for study personnel. *Trials* **20,** 618 (2019). https://doi.org/10.1186/s13063-019-3737-1

53. Caldwell PH, Hamilton S, Tan A et al. Strategies for increasing recruitment to randomised controlled trials: systematic review. *PLoS Medicine 2010; 7*(11):e1000368. https://doi.org/10.1371/journal.pmed.1000368

54. Öberg M. A multicentre study evaluating the effects of the Swedish ACE programme. *Int J Audiol* 2017;56: 876-886.

For peer review only - http://bmjopen.bmj.com/site/about/guidelines.xhtml

Figure 1: The flow of participants through the trial shown in a CONSORT diagram

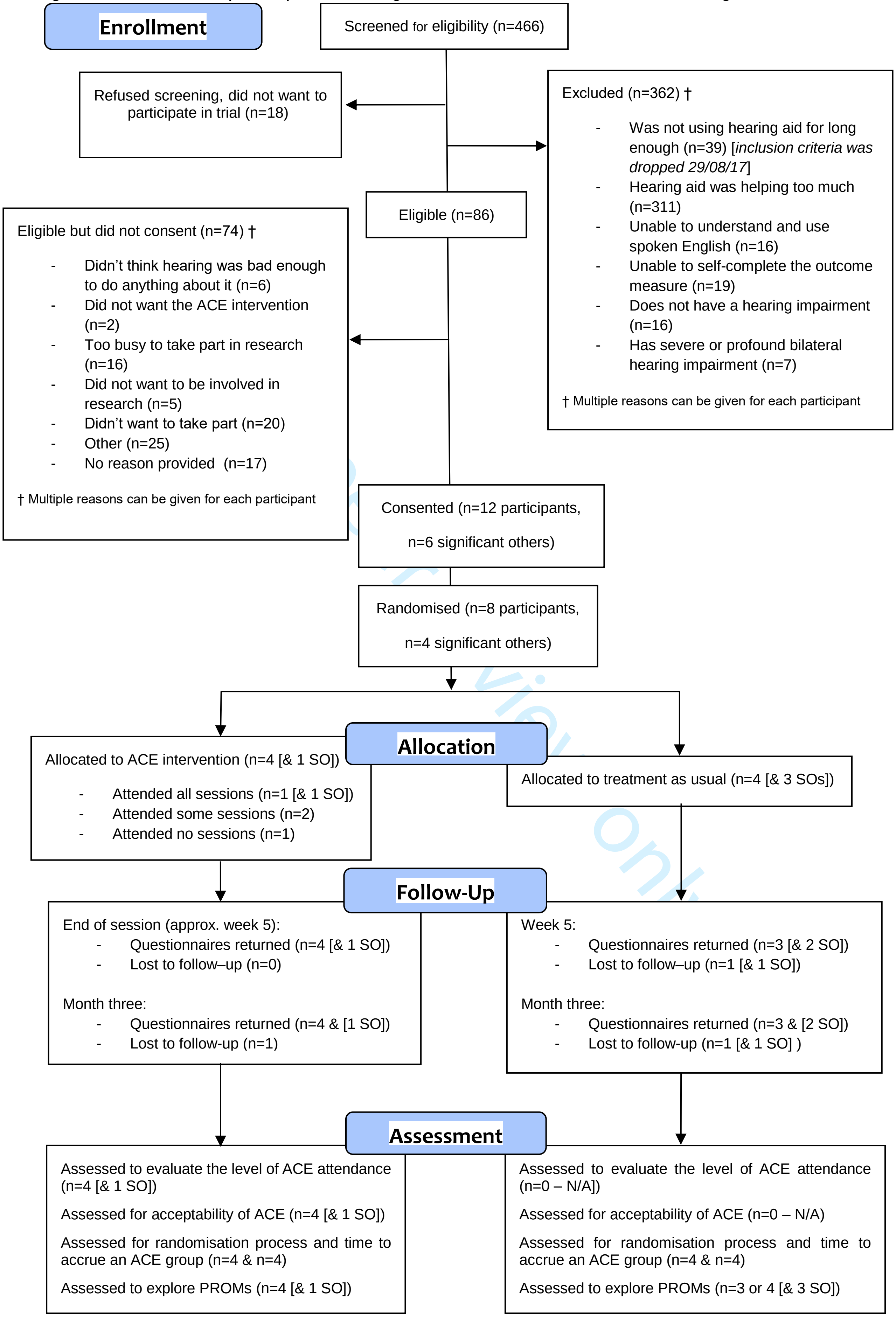

Supplementary material for manuscript:

A randomised controlled feasibility trial of an Active Communication Education programme plus hearing aid provision versus hearing aid provision alone (ACE To HEAR)

Figure A: Monthly recruitment to the study, by site

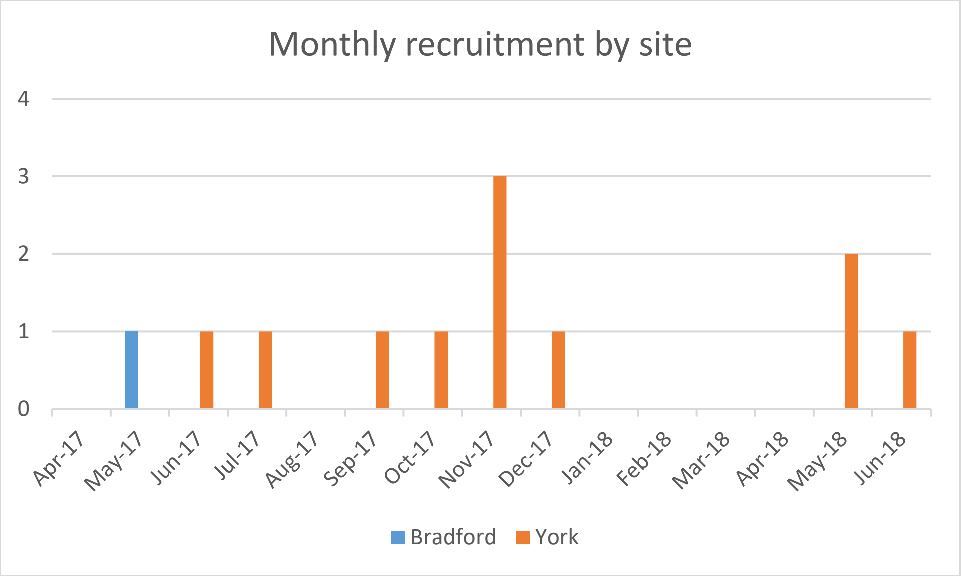

Table A: Hearing aid use, hearing thresholds and aetiology for the randomised participants by group and overall

	**ACE Intervention (n=4)**	**Control (n=4)**	**All (n=8)**
**Average daily hearing aid use over the past two weeks:**			
None	1 (25.0)	0 (0.0)	1 (12.5)
Less than 1 hour a day	0 (0.0)	0 (0.0)	0 (0.0)
Between 1 and 4 hours a day	1 (25.0)	3 (75.0)	4 (50.5)
Between 4 and 8 hours a day	0 (0.0)	0 (0.0)	0 (0.0)
More than 8 hours a day	2 (50.0)	1 (25.0)	3 (37.5)

**Level of help from hearing aid in the last two weeks:**			
Helped not at all	1 (25.0)	0 (0.0)	1 (12.5)
Helped slightly	1 (25.0)	1 (25.0)	2 (25.0)
Helped moderately	2 (50.0)	3 (75.0)	5 (62.5)
Helped quite a lot	0 (0.0)	0 (0.0)	0 (0.0)
Helped very much	0 (0.0)	0 (0.0)	0 (0.0)
Hearing Thresholds			
	N=4	N=4	N=8
Left Ear	27.5 (9.6)	18.8 (8.5)	23.1 (9.6)
**500Hz**	30 (15, 35)	17.5 (10, 30)	22.5 (10, 35)
Mean (SD)			
Median (min, max)			
	40 (20.4)	26.3 (13.8)	33.1 (17.7)
**1000Hz**	40 (15, 65)	27.5 (10, 40)	37.5 (10, 65)
Mean (SD)			
Median (min, max)			
	61.3 (11.1)	41.3 (8.5)	51.3 (14.1)
**2000Hz**	60 (50, 75)	42.5 (30, 50)	50 (30, 75)
Mean (SD)			
Median (min, max)			
	75 (9.1)	52.5 (10.4)	63.8 (15.1)
**4000Hz**	75 (65, 85)	52.5 (40, 65)	65 (40, 85)
Mean (SD)			
Median (min, max)			
	73.8 (23.2)	70 (9.2)	71.9 (16.5)
**8000Hz**	77.5 (45, 95)	70 (60, 80)	70 (45, 95)
Mean (SD)			
Median (min, max)			
Right Ear			
**500Hz**	N=4	N=4	N=8
Mean (SD)	26.3 (18.9)	22.5 (8.7)	24.4 (13.7)
Median (min, max)	25 (10, 45)	20 (15, 35)	20 (10, 45)
**1000Hz**			
Mean (SD)	36.3 (15.5)	32.5 (15.5)	34.4 (14.5)
Median (min, max)	40 (15, 50)	37.5 (10, 45)	37.5 (10, 50)
**2000Hz**			
Mean (SD)	41.3 (7.5)	52.5 (22.2)	46.9 (16.5)
Median (min, max)	45 (30, 45)	50 (30, 80)	45 (30, 80)
**4000Hz**			

For peer review only

Mean (SD)<br>Median (min, max)	75 (14.8)<br>75 (60, 90)	63.8 (19.3)<br>62.5 (45, 85)	69.4 (17.0)<br>70 (45, 90)
**8000Hz**<br>Mean (SD)<br>Median (min, max)	80 (23.8)<br>90 (45, 95)	71.3 (25.0)<br>65 (50, 105)	75.6 (23.1)<br>80 (45, 105)
**Aetiology, n (%)**			
Sensorineural	3 (75.0)	4 (100.0)	7 (87.5)
Conductive	0 (0.0)	0 (0.0)	0 (0.0)
Mixed	1 (25.0)	0 (0.0)	1 (12.5)
Other	0 (0.0)	0 (0.0)	0 (0.0)

Table B: International Outcomes Inventory for Hearing Aids (IOI-HA) results for participants, by group and overall (Higher score is better, range 7-35)

	ACE Intervention participants (n=4)	Control participants (n=4)	All participants (n=8)
**Baseline**<br>Mean (SD)<br>Median (min, max)	N=4<br>22.5 (6.6)<br>23 (15, 29)	N=3<br>25.7 (3.1)<br>25 (23, 29)	N=7<br>23.9 (5.3)<br>25 (15, 29)
**Week 5**<br>Mean (SD)<br>Median (min, max)	N=3<br>23.0 (3.6)<br>24 (19, 26)	N=3<br>26.3 (4.7)<br>28 (21, 30)	N=6<br>24.7 (4.2)<br>25 (19, 30)
**3 months post-randomisation**<br>Mean (SD)<br>Median (min, max)	N=3<br>20 (1.7)<br>19 (19, 22)	N=3<br>27.7 (3.5)<br>28 (24, 31)	N=6<br>23.9 (4.9)<br>23 (19, 31)

Table C: Self-Assessment of Communication (SAC) (Lower score is better, range 0 – 100)

	ACE Intervention participants (n=4)	Control Participants (n=4)	All participants (n=8)
**Baseline**<br>Mean (SD)<br>Median (min, max)	N=4<br>65 (21.9)<br>58.8 (47.5, 95)	N=4<br>20 (7.9)<br>21.3 (10, 27.5)	N=8<br>42.5 (28.5)<br>37.5 (10, 95)
**3 months post-randomisation**<br>Mean (SD)<br>Median (min, max)	N=3<br>62.5 (10.9)<br>57.5 (55, 75)	N=3<br>22.5 (4.3)<br>25 (17.5, 25)	N=6<br>42.5 (23.1)<br>40 (17.5, 75)

For peer review only

Table D: International Outcomes Inventory for Alternative Interventions (IOI-AI) (scored 1 – 5, higher score is better)

	ACE intervention participants (n=4)
**Week 5**	N=3
Mean (SD)	2.6 (0.7)
Median (min, max)	3 (1.9, 3.1)
**3 months post-randomisation**	N=2
Mean (SD)	2.6 (0.6)
Median (min, max)	2.6 (2.1, 3)

Table E: International Outcomes Inventory for Hearing Aids: version for Significant Others (IOI-HA-SO) results for significant others, overall (Higher score is better, range 7 - 35)

	All SOs (n=4)
**Baseline**	N=4
Mean (SD)	23.8 (5.3)
Median (min, max)	24 (17, 30)
**Week 5**	N=3
Mean (SD)	25.7 (3.1)
Median (min, max)	25 (23, 29)
**3 months post-randomisation**	N=3
Mean (SD)	24 (4.0)
Median (min, max)	24 (20, 28)

Table F: Significant Other Scale for Hearing Disability (SOS-HEAR) (scored 0 – 4, higher scores indicate greater difficulties experienced by the SO.)

	All SOs (n=4)
**Baseline**	N=4
Mean (SD)	1.0 (0.5)
Median (min, max)	0.9 (0.5, 1.8)
**Week 5**	N=3
Mean (SD)	1.2 (1.0)
Median (min, max)	0.8 (0.5, 2.3)
**3 months post-randomisation**	N=3
Mean (SD)	1.4 (1.0)
Median (min, max)	1.1 (0.6, 2.4)

For peer review only

Table G: Average health care resource use by trial arm at baseline

	ACE				TAU			
	n	Mean (SD)	Min, Max	Missing	n	Mean (SD)	Min, Max	Missing
**GP Visit**	2	0.5 0.71	0 1	2	4	0	0, 0	0
**home**	2	0 (0)	0, 0	2	4	0 (0)	0, 0	0
**Physiotherapist**	2	0 (0)	0, 0	2	4	0 (0)	0, 0	0
**Nurse**	2	0 (0)	0, 0	2	4	0 (0)	0, 0	0
**District/ community nurse**	0	0 (0)	0, 0		4	0 (0)	0, 0	0
**Hearing therapist**	2	1 (1.41)	0, 2	2	3	0.33 (0.58)	0, 1	1
**Speech therapist**	2	0 (0)	0, 0	2	4	0 (0)	0, 0	0
**Counsellor**	2	0 (0)	0, 0	2	4	0 (0)	0, 0	0
**Psychologist**	2	0 (0)	0, 0	2	4	0 (0)	0, 0	0
**Psychiatrist**	2	0 (0)	0, 0	2	4	0 (0)	0, 0	0
**ENT nurse**	2	0 (0)	0, 0	2	4	0.75 (1.5)	0, 3	0
**ENT doctor**	2	0 (0)	0, 0	2	4	0.75 (1.5)	0, 3	0
**Outpatient attendance**	2	1.5 (0.71)	1 2	2	4	1.75 (1.71)	0, 4	0
**A&E attendance**	2	0 (0)	0, 0	2	4	0 (0)	0, 0	0
**Day case**	2	0.5 (0.71)	0, 1	2	4	0 (0)	0, 0	0
**In patient per night**	2	0 (0)	0, 0	2	4	0 (0)	0, 0	0
**Private physiotherapist**	2	0 (0)	0, 0	2	4	0 (0)	0, 0	0
**Private audiologist**	2	0 (0)	0, 0	2	4	0 (0)	0, 0	0
**Private osteopath**	2	0 (0)	0, 0	2	4	0 (0)	0, 0	0
**Private counsellor**	2	0 (0)	0, 0	2	4	0 (0)	0, 0	0
**Private acupuncturist**	2	0 (0)	0, 0	2	4	0 (0)	0, 0	0
**Private chiropractitioner**	2	0 (0)	0, 0	2	4	0 (0)	0, 0	0
**Private day case**	2	0 (0)	0, 0	2	4	0 (0)	0, 0	0
**Private in patient per night**	2	0 (0)	0, 0	2	4	0 (0)	0, 0	0

For peer review only

Table H: Average health care resource use by trial arm at 3 months post-randomisation

	ACE				TAU			
	**n**	**Mean (SD)**	**Min, Max**	**Missing**	**n**	**Mean (SD)**	**Min, Max**	**Missing**
**GP Visit**	1	0 (0)	0, 0	3	3	0 (0)	0, 0	1
**home**	1	0 (0)	0, 0	3	3	0 (0)	0, 0	1
**Physiotherapist**	1	0 (0)	0, 0	3	3	0 (0)	0, 0	1
**Nurse**	0	-	-	4	3	0 (0)	0, 0	1
**District/ community nurse**	1	0 (0)	0, 0	3	3	0 (0)	0, 0	1
**Hearing therapist**	2	0.5 (0.71)	0, 1	2	3	0 (0)	0, 0	1
**Speech therapist**	1	0 (0)	0, 0	3	3	0 (0)	0, 0	1
**Counsellor**	1	0 (0)	0, 0	3	3	0 (0)	0, 0	1
**Psychologist**	1	0 (0)	0, 0	3	3	0 (0)	0, 0	1
**Psychiatrist**	1	0 (0)	0, 0	3	3	0 (0)	0, 0	1
**ENT nurse**	1	1 (-)	1, 1	3	3	0 (0)	0, 0	1
**ENT doctor**	1	0 (0)	0, 0	3	3	0 (0)	0, 0	1
**Outpatient attendance**	1	1 (-)	1, 1	3	3	0 (0)	0, 0	1
**A&E attendance**	1	0 (0)	0, 0	3	3	0 (0)	0, 0	1
**Day case**	1	0 (0)	0, 0	3	3	0 (0)	0, 0	1
**In patient per night**	1	0 (0)	0, 0	3	3	0 (0)	0, 0	1
**Private physiotherapist**	1	0 (0)	0, 0	3	3	0 (0)	0, 0	1
**Private audiologist**	1	2 (-)	2, 2	3	3	0 (0)	0, 0	1
**Private osteopath**	1	0 (0)	0, 0	3	3	0 (0)	0, 0	1
**Private counsellor**	1	0 (0)	0, 0	3	3	0 (0)	0, 0	1
**Private acupuncturist**	1	0 (0)	0, 0	3	3	0 (0)	0, 0	1
**Private chiropractitioner**	1	0 (0)	0, 0	3	3	0 (0)	0, 0	1
**Private day case**	1	0 (0)	0, 0	3	3	0 (0)	0, 0	1
**Private in patient per night**	1	0 (0)	0, 0	3	3	0 (0)	0, 0	1

For peer review only

CONSORT 2010 checklist of information to include when reporting a pilot or feasibility randomized trial in a journal or conference abstract

Item	Description	Reported on manuscript line number
Title	Identification of study as randomised pilot or feasibility trial	1
Authors *	Contact details for the corresponding author	28
Trial design	Description of pilot trial design (eg, parallel, cluster)	36-37
Methods		
Participants	Eligibility criteria for participants and the settings where the pilot trial was conducted	38; 39-40
Interventions	Interventions intended for each group	41-43
Objective	Specific objectives of the pilot trial	33-35
Outcome	Prespecified assessment or measurement to address the pilot trial objectives**	44-46
Randomization	How participants were allocated to interventions	42
Blinding (masking)	Whether or not participants, care givers, and those assessing the outcomes were blinded to group assignment	48-49
Results		
Numbers randomized	Number of participants screened and randomised to each group for the pilot trial objectives**	50-51; 53
Recruitment	Trial status†	NA
Numbers analysed	Number of participants analysed in each group for the pilot objectives**	50-51
Outcome	Results for the pilot objectives, including any expressions of uncertainty**	55-56
Harms	Important adverse events or side effects	Main text 353
Conclusions	General interpretation of the results of pilot trial and their implications for the future definitive trial	57-59
Trial registration	Registration number for pilot trial and name of trial register	60
Funding	Source of funding for pilot trial	Main text 560

Citation: Eldridge SM, Chan CL, Campbell MJ, Bond CM, Hopewell S, Thabane L, et al. CONSORT 2010 statement: extension to randomised pilot and feasibility trials. BMJ. 2016;355.

**this item is specific to conference abstracts*

***Space permitting, list all pilot trial objectives and give the results for each. Otherwise, report those that are a priori agreed as the most important to the decision to proceed with the future definitive RCT.*

†For conference abstracts.

For peer review only

CONSORT 2010 checklist of information to include when reporting a pilot or feasibility trial*

Section/Topic	Item No	Checklist item	Reported on page No
**Title and abstract**			
	1a	Identification as a pilot or feasibility randomised trial in the title	1
	1b	Structured summary of pilot trial design, methods, results, and conclusions (for specific guidance see CONSORT abstract extension for pilot trials)	3
**Introduction**			
Background and objectives	2a	Scientific background and explanation of rationale for future definitive trial, and reasons for randomised pilot trial	5-6
	2b	Specific objectives or research questions for pilot trial	6-7
**Methods**			
Trial design	3a	Description of pilot trial design (such as parallel, factorial) including allocation ratio	6
	3b	Important changes to methods after pilot trial commencement (such as eligibility criteria), with reasons	7-8
Participants	4a	Eligibility criteria for participants	3; 7
	4b	Settings and locations where the data were collected	6
	4c	How participants were identified and consented	6
Interventions	5	The interventions for each group with sufficient details to allow replication, including how and when they were actually administered	6
Outcomes	6a	Completely defined prespecified assessments or measurements to address each pilot trial objective specified in 2b, including how and when they were assessed	8-10
	6b	Any changes to pilot trial assessments or measurements after the pilot trial commenced, with reasons	-
	6c	If applicable, prespecified criteria used to judge whether, or how, to proceed with future definitive trial	9-10
Sample size	7a	Rationale for numbers in the pilot trial	7
	7b	When applicable, explanation of any interim analyses and stopping guidelines	-
Randomisation:			
Sequence generation	8a	Method used to generate the random allocation sequence	8
	8b	Type of randomisation(s); details of any restriction (such as blocking and block size)	8
Allocation concealment mechanism	9	Mechanism used to implement the random allocation sequence (such as sequentially numbered containers), describing any steps taken to conceal the sequence until interventions were assigned	8

For peer review only

Implementation	10	Who generated the random allocation sequence, who enrolled participants, and who assigned participants to interventions	8
Blinding	11a	If done, who was blinded after assignment to interventions (for example, participants, care providers, those assessing outcomes) and how	3
	11b	If relevant, description of the similarity of interventions	-
Statistical methods	12	Methods used to address each pilot trial objective whether qualitative or quantitative	10-11
**Results**			
Participant flow (a diagram is strongly recommended)	13a	For each group, the numbers of participants who were approached and/or assessed for eligibility, randomly assigned, received intended treatment, and were assessed for each objective	Fig 1; p12
	13b	For each group, losses and exclusions after randomisation, together with reasons	12-13
Recruitment	14a	Dates defining the periods of recruitment and follow-up	12
	14b	Why the pilot trial ended or was stopped	-
Baseline data	15	A table showing baseline demographic and clinical characteristics for each group	14
Numbers analysed	16	For each objective, number of participants (denominator) included in each analysis. If relevant, these numbers should be by randomised group	13-19
Outcomes and estimation	17	For each objective, results including expressions of uncertainty (such as 95% confidence interval) for any estimates. If relevant, these results should be by randomised group	13-22
Ancillary analyses	18	Results of any other analyses performed that could be used to inform the future definitive trial	13-22
Harms	19	All important harms or unintended effects in each group (for specific guidance see CONSORT for harms)	19
	19a	If relevant, other important unintended consequences	
**Discussion**			
Limitations	20	Pilot trial limitations, addressing sources of potential bias and remaining uncertainty about feasibility	22-27
Generalisability	21	Generalisability (applicability) of pilot trial methods and findings to future definitive trial and other studies	22-27
Interpretation	22	Interpretation consistent with pilot trial objectives and findings, balancing potential benefits and harms, and considering other relevant evidence	22-27
	22a	Implications for progression from pilot to future definitive trial, including any proposed amendments	27
**Other information**			
Registration	23	Registration number for pilot trial and name of trial registry	4
Protocol	24	Where the pilot trial protocol can be accessed, if available	6
Funding	25	Sources of funding and other support (such as supply of drugs), role of funders	28
	26	Ethical approval or approval by research review committee, confirmed with reference number	28

For peer review only

Citation: Eldridge SM, Chan CL, Campbell MJ, Bond CM, Hopewell S, Thabane L, et al. CONSORT 2010 statement: extension to randomised pilot and feasibility trials. BMJ. 2016;355.

*We strongly recommend reading this statement in conjunction with the CONSORT 2010, extension to randomised pilot and feasibility trials, Explanation and Elaboration for important clarifications on all the items. If relevant, we also recommend reading CONSORT extensions for cluster randomised trials, non-inferiority and equivalence trials, non-pharmacological treatments, herbal interventions, and pragmatic trials. Additional extensions are forthcoming: for those and for up to date references relevant to this checklist, see www.consort-statement.org.

For peer review only
